# Efficient scalable production of therapeutic microvesicles derived from human mesenchymal stem cells

**DOI:** 10.1038/s41598-018-19211-6

**Published:** 2018-01-19

**Authors:** Jae Min Cha, Eun Kyoung Shin, Ji Hee Sung, Gyeong Joon Moon, Eun Hee Kim, Yeon Hee Cho, Hyung Dal Park, Hojae Bae, Jinseok Kim, Oh Young Bang

**Affiliations:** 1 Medical Device Research Centre, Research Institute for Future Medicine, Samsung Medical Centre, Seoul, 06351 Republic of Korea; 2 Translational and Stem Cell Research Laboratory on Stroke, Samsung Medical Centre, Seoul, 06351 Republic of Korea; 3 Stem Cell and Regenerative Medicine Centre, Research Institute for Future Medicine, Samsung Medical Centre, Seoul, 06351 Republic of Korea; 4 Centre for Bionics of Biomedical Research Institute, Korea Institute of Science and Technology, Seoul, 03722 Republic of Korea; 5 Department of Mechanical Engineering, Yonsei University, Seoul, 02792 Republic of Korea; 6 KU Convergence Science and Technology Institute, Department of Stem Cell and Regenerative Biology, Konkuk University, Seoul, 05029 Republic of Korea; 7 Department of Neurology, Samsung Medical Centre, School of Medicine, Sungkyunkwan University, Seoul, 06351 Republic of Korea

## Abstract

Microvesicles (MVs) released by cells are involved in a multitude of physiological events as important mediators of intercellular communication. MVs derived from mesenchymal stem cells (MSCs) contain various paracrine factors from the cells that primarily contribute to their therapeutic efficacy observed in numerous clinical trials. As nano-sized and bi-lipid layered vesicles retaining therapeutic potency equivalent to that of MSCs, MSC-derived MVs have been in focus as ideal medicinal candidates for regenerative medicine, and are preferred over MSC infusion therapy with their improved safety profiles. However, technical challenges in obtaining sufficient amounts of MVs have limited further progress in studies and clinical application. Of the multiple efforts to reinforce the therapeutic capacity of MSCs, few studies have reportedly examined the scale-up of MSC-derived MV production. In this study, we successfully amplified MV secretion from MSCs compared to the conventional culture method using a simple and efficient 3D-bioprocessing method. The MSC-derived MVs produced in our dynamic 3D-culture contained numerous therapeutic factors such as cytokines and micro-RNAs, and showed their therapeutic potency in *in vitro* efficacy evaluation. Our results may facilitate diverse applications of MSC-derived MVs from the bench to the bedside, which requires the large-scale production of MVs.

## Introduction

**Figure 1:**
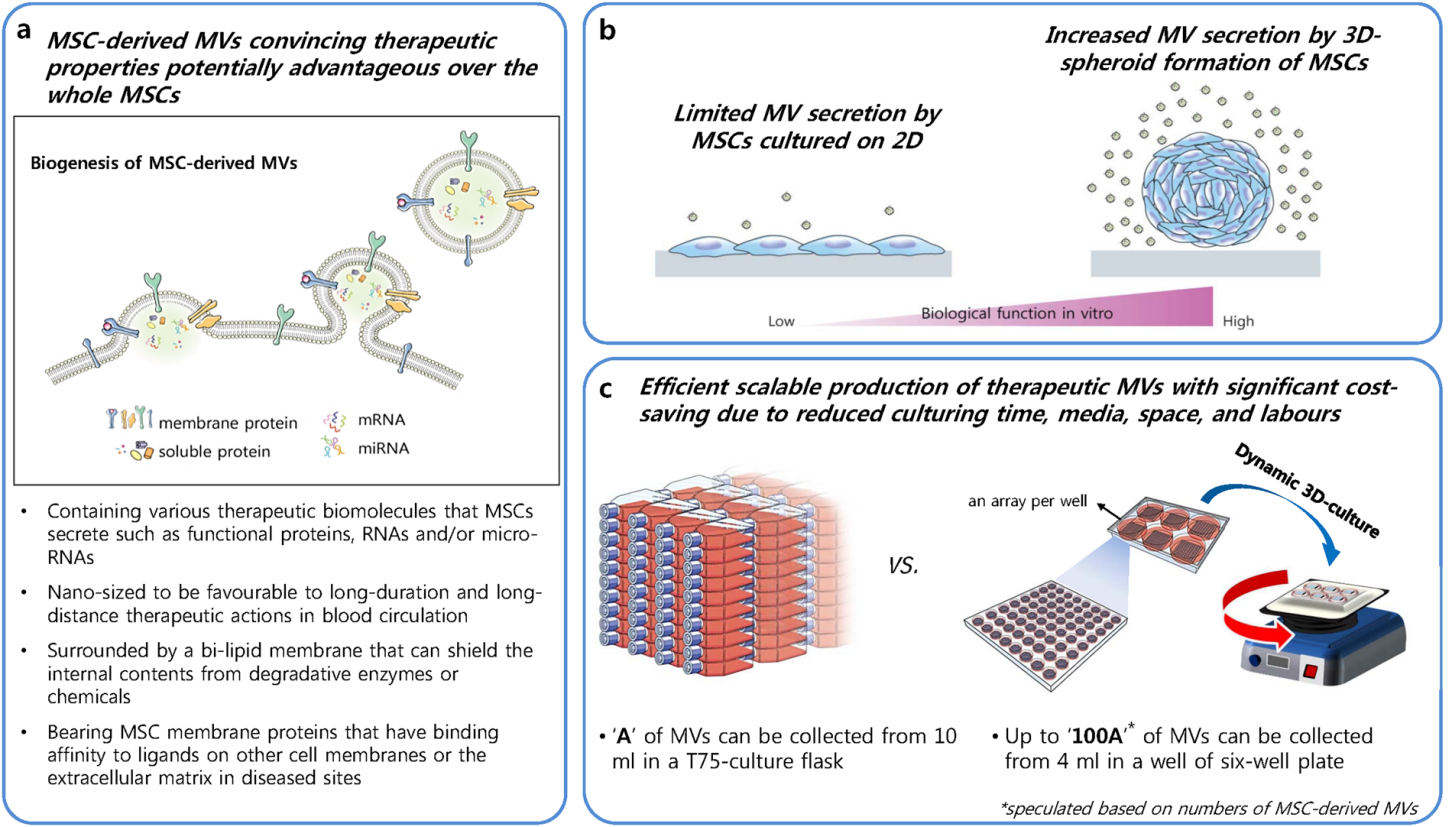
Schematic summary of the study. (**a**) MSC-derived MVs are a promising therapeutic tool, with several advantages over current MSC-therapies and soluble biomolecular medications. (**b**) 3D MSC-culture can address the technical challenges facing conventional culture methods to obtain sufficient amounts of MSC-derived MVs for research and clinical use. (**c**) Scalable production of therapeutic MSC-derived MVs can be achieved by the simple and effectual 3D MSC-bioprocessing method presented in this study.

Currently, numerous clinical trials of mesenchymal stem cells (MSCs) are being conducted to develop effective treatments for diseases with few curable options, such as stroke, spinal cord injury, multiple sclerosis, Alzheimer’s disease, liver cirrhosis, myocardial infarction, kidney disease, and graft-versus-host disease, among others. (www.clinicaltrials.gov). Although positive clinical outcomes have been demonstrated in many cases, current approaches used for MSC transplantation therapy are challenging due to limited cell sources, difficulty in administration during an optimal time window for stem cell treatment, entrapment in undesired organs/tissues on systemic injection, vascular occlusion because of the relatively large cell size, and possibility of forming tumours or undesirable ossification/calcification in tissues, raising long-term safety concerns^1–6^. While increasing evidence has shown that the clinical efficacy of MSCs is mainly attributed to their paracrine effects, microvesicles (MVs) released by MSCs have gained attention for their paracrine communication ability in the field of regenerative medicine^7–9^. MSC-derived MVs possess the following crucial properties required for a cell-free therapeutic strategy as a substitute for current MSC transplantation therapy: 1) they contain numerous therapeutic biomolecules released by MSCs, 2) their nano-sized and lipid-shielded vesicular structure would be safer and more favourable for long-duration in blood circulation and long-distance therapeutic actions than MSCs, and 3) MSC membrane proteins on their surfaces may confer disease-targeting ability such as those of infused MSCs (Fig. 1a)^10–12^. Furthermore, MVs have potential advantages over live MSCs with respect to biomanufacturing, storing/shipping, and administering MVs, such as the feasibility of continuous production with culturing of MSCs, sustained stability in the regular freezing process at −80 °C, and few detrimental effects in the thawing and clinical administration processes^13–15^.

However, a lack of bioprocessing methods for scaling up the derivation of MVs from MSCs is one of the major limitations to the use of this promising medicinal product for both research and clinical purposes. As reported by numerous previous studies, the intrinsic ability of MSCs to secrete a variety of therapeutic molecules is difficult to reproduce *in vitro*, as natural 3D-interactions between cells and either the extracellular matrix (ECM) or other neighbouring cells are readily disrupted under conventional monolayer conditions where individual cells encounter limited two-dimensional borders^16,17^. However, MSCs in self-assembled aggregates are in a three-dimensionally closer association with each other, ensuring improved cellular communication with highly cumulated signalling molecules compared to in monolayer cultures. Numerous recent studies have reported that the formation of 3D MSC-aggregates can create a microenvironment similar to that *in vivo* where the phenotype and innate properties of the MSC are highly preserved^18,19^. Thus, we hypothesized that the formation of MSC-spheroids would enhance the production of therapeutic MVs in subsequent 3D-culture (Fig. 1b). In this study, we successfully amplified therapeutic MSC-derived MVs by using a simple and effectual dynamic 3D-culture method (Fig. 1c). Size-controlled human MSC (hMSC) aggregates were homogeneously formed on a large scale by using a polyethylene glycol (PEG) hydrogel microwell array modified from that reported in our previous studies^20,21^. By subsequently providing a dynamic culture environment, the production of MSC-derived MVs was significantly increased compared to that using a conventional monolayer culture method. Mass-produced MVs were comprehensively characterized as nano-sized and lipid-membranous vesicles, showing similar results to those of previous studies. The inclusion of a variety of therapeutic factors in MSC-derived MVs from our dynamic 3D-culture was investigated, and their medicinal potency was evaluated using different culture models.

## Results

### Formation and culture of size-controlled hMSC-spheroids

**Figure 2:**
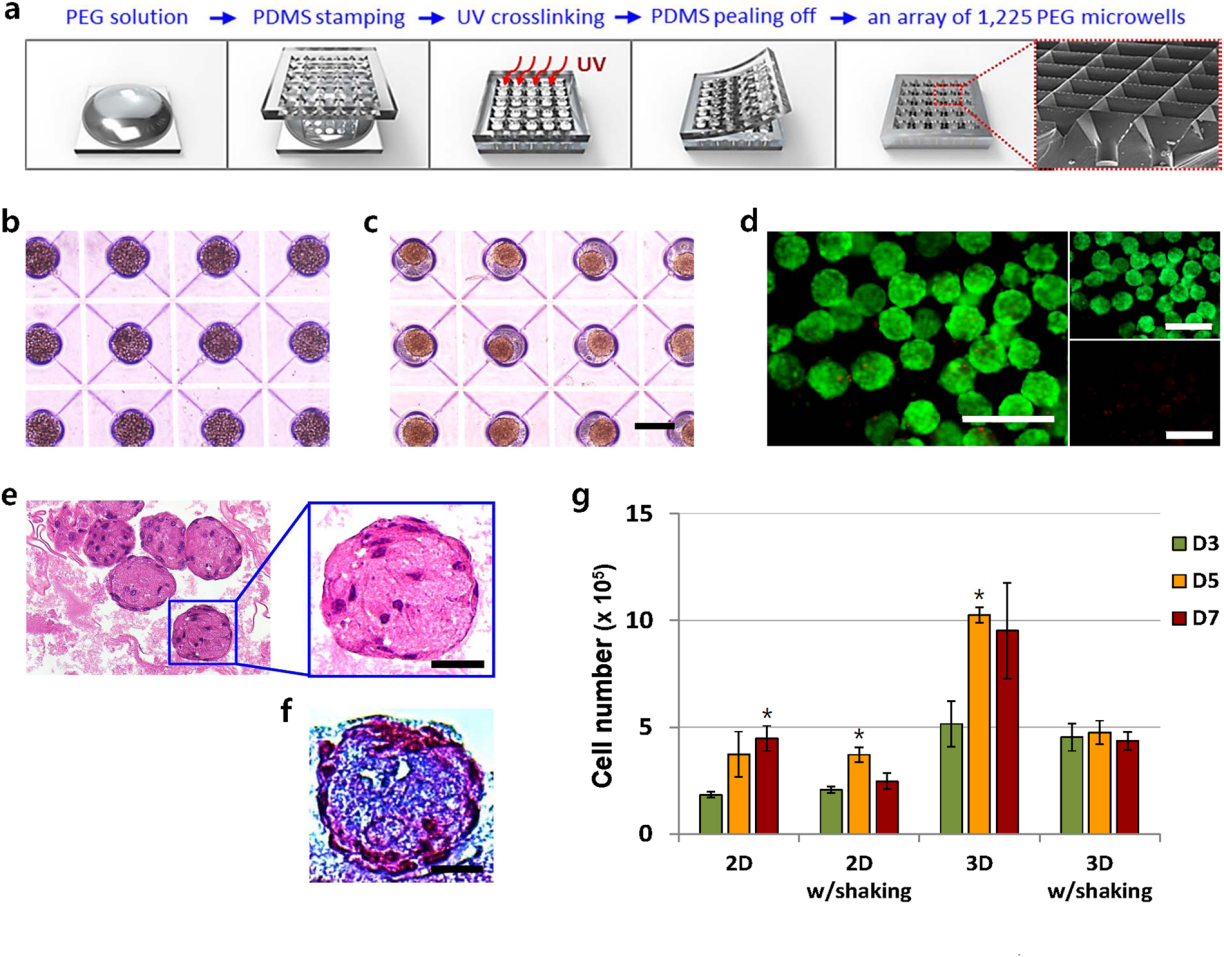
Large-scale formation of hMSC-spheroids with precisely controlled size and cell number. (**a**) Fabrication of a PEG hydrogel microwell array with inverted-pyramidal openings adjoined to cylindrical microwells. (**b**) Seeded hMSCs, at a density of 5 × 10^5^ cells/array, were evenly entrapped within microwells 20 min after seeding. (**c**) 12 hours after cell seeding, hMSCs entrapped within microwells were well agglomerated in the shape of a spheroid with a controlled size of approximately 150 μm. The size bar indicates 200 μm. The microwell arrays were inserted in the commercial six-well plates and cultured for 7 days in a CO_2_ incubator under a 30-rpm orbital-shaking condition. (**d**) A live (green) and dead (red) assay of the 3D w/shaking group on D5 revealed that most cells in the 3D hMSC-aggregates were highly viable. The size bar indicates 400 μm. (**e** and **f**) Histological images after H&E (**e**) and M&T (**f**) staining showed that hMSC-spheroids of the 3D w/shaking group on D5 were compactly integrated with cells and secreted ECM, respectively. The size bar indicates 50 μm. (**g**) Cell growth kinetics was examined by a DNA quantification method. The cell numbers in the 3D w/shaking group were not increased during the culture period from the initial seeding density. Data are presented as the mean ± SEM. Differences among culture days in each group were evaluated by one-way ANOVA at a level of significance of p < 0.05 (*).

Large-scale formation of hMSC-spheroids was accomplished using a PEG hydrogel microwell array modified from previous studies^20–22^. Our custom-engineered microwell array was composed of cylindrical microwells with inverted-pyramidal openings (Fig. 2a), which successfully prevented cell loss during the mass-production of hMSC-spheroids. In addition, our optimized PEG hydrogel soft-lithography techniques achieved complete resistance to cellular adhesion on the microwell substrate. Because all seeded hMSCs could be used to form hMSC-spheroids, the sizes and cell numbers of resultant spheroids were precisely controlled with high consistency. The dimensions of our microwell array containing 1,225 microwells, each 200 μm in diameter, were 20 × 20 mm to fit into a well of a commercial six-well plate. The hMSCs were seeded at a density of 5 × 10^5^ cells/array (~400 cells/microwell) and spontaneously formed spherical cellular aggregates within 12 hours (Fig. 2b and c). The hMSC-spheroids were homogeneously sized with a diameter of approximately 150 μm, which was smaller than the size of the microwell, because of the structural compaction that cellular aggregates typically undergo^23^. hMSC-spheroids were subsequently cultured for 7 days on a 30-rpm orbital-shaker (3D w/shaking). A live and dead assay of the 3D w/shaking group showed that most cells in the 3D-aggregates were highly viable during the culture period (Fig. 2d and Supplementary Figure 1). Histological staining with haematoxylin and eosin (H&E) and Masson’s trichrome (M&T) revealed that hMSC-spheroids were compactly integrated with cells and secreted ECM components (Fig. 2e and f). The cell growth kinetics of the 3D w/shaking group was examined using a DNA quantification method and compared with three other culture conditions, monolayer cultures without or with orbital-shaking (2D or 2D w/shaking) and 3D culture without orbital-shaking (3D) (Fig. 2g). The 2D group displayed the expected hMSC expansion rate that increased with culture time, whereas cells in the 2D w/shaking group decreased after day (D) 5, possibly because of the vulnerability of attached cells to the shear stress exerted continuously during the shaking condition based on microscopy analysis (Supplementary Figure 2). After formation in the microwells, hMSC-spheroids in the 3D group were transferred to a petri-dish and cultured without shaking. Interestingly, although the petri-dish was designed to be non-adhesive to cells, hMSC-spheroids attached on the substrate after D1 and spread to surrounding areas (Supplementary Figure 2). It was previously reported that 3D-cultured MSCs showed significantly reinforced ECM secretion compared to those cultured on a monolayer^23^. Therefore, we speculated that the extensive secretion of ECM molecules by hMSC-spheroids initially spread across the petri-dish and subsequently hMSCs in the 3D group may have attached over them. The growth kinetics of the 3D group revealed that the cell number nearly doubled by D5, reaching confluence with cell growth on D7. However, the cell numbers in our 3D w/shaking group showed no increase from the initial seeding density over the culture period. This result agreed with those of previous studies reporting that MSC-spheroids cultured in suspension using foetal bovine serum (FBS)-containing medium maintained their initial cell numbers, while their biological properties as stem cells were enhanced^24,25^.

### Gene expression profiles of hMSC-spheroids grown by dynamic 3D-culture

**Figure 3:**
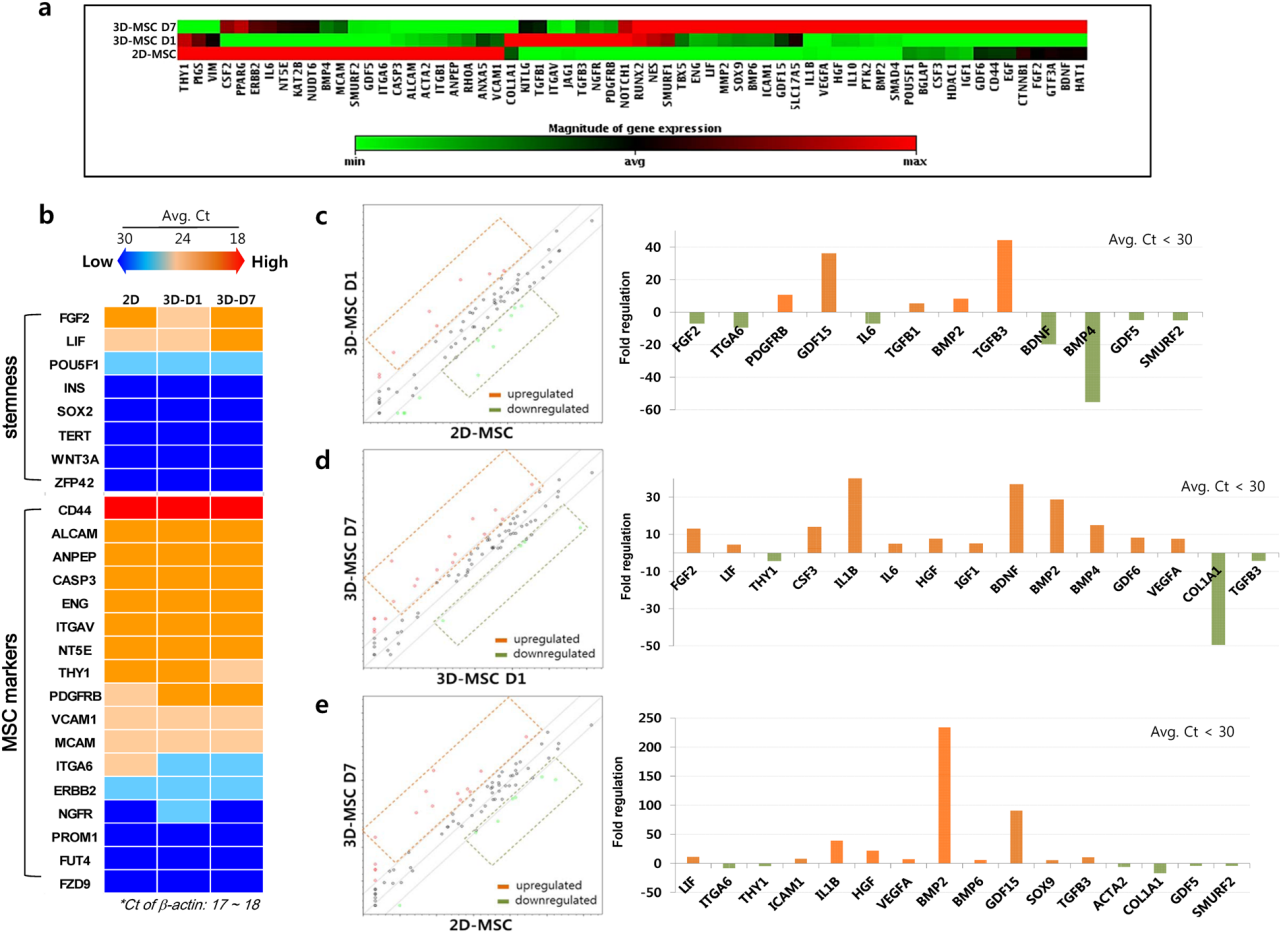
Gene expression profiles of hMSC-spheroids grown in dynamic 3D-culture. (**a**) A cluster gram of the PCR array displayed the expression profile of 84 key genes related to the nature of hMSCs, which relatively differed between the 2D- and 3D-MSCs. The gene expression profile of the 3D-MSCs on D1 appeared to be transitional, as they were in the process of forming 3D-spheroids during the 7-day culture period. (**b**) Comparison of gene expression related to stemness and MSC markers. *FGF2*, *LIF*, and *POU5F1* were expressed in all groups, while *FGF2* and *LIF* generally decreased upon formation of hMSC-spheroids and subsequently increased over culture time. A variety of hMSC marker genes was highly expressed in 3D-MSCs, showing levels and patterns comparable to those in 2D-MSCs. (**c**–**e**) Gene expression representing hMSC’s attributes with average Ct values below 30 are presented by scatter plots and compared between groups. Upon formation of hMSC-spheroids (D1), *GDF15* and *TGFB3* were upregulated by approximately 40-fold compared to the 2D control, whereas *BMP4* was downregulated by approximately 60-fold (**c**). As our dynamic 3D hMSC-culture progressed up to D7, *IL1B*, *BDNF*, and *BMP2* were upregulated by more than 30-fold while *COL1A1* was downregulated by approximately 50-fold, compared to the early stage of 3D-MSCs on D1 (**d**). Comparison between 3D-MSC on D7 and 2D-MSCs showed that *IL1B* and *GDF15* were upregulated by approximately 40- and 90-fold, respectively. Particularly, *BMP2* was extensively upregulated by approximately 230-fold (**e**).

Gene expression profiling was carried out to differentiate between the stem cell characteristics of 2D- and dynamic 3D-MSCs using a PCR array to examine 84 key genes related to the generic nature of hMSCs. A cluster gram of the PCR array was used to display the expression profile of the genes, which differed relatively between the 2D- and dynamic 3D-MSCs (Fig. 3a). The gene expression profile of dynamic 3D-MSCs on D1 appeared to be transitional, as these cells were in the process of forming 3D-spheroids during the 7 days of culture. Genes related to stemness, such as *FGF2*, *LIF*, and *POU5F1*, were expressed in all groups, while *FGF2* and *LIF* generally decreased during the formation of hMSC-spheroids and subsequently increased with the following 3D-culture. A variety of hMSC marker genes was highly expressed in dynamic 3D-MSCs with levels and patterns comparable to those in 2D-MSCs (Fig. 3b).

Gene expressions implicating hMSC’s attributes with average Ct values below 30 were presented in scatter plots and compared between groups. To avoid overestimating assessments, upregulation or downregulation by less than 30-fold was not considered as a meaningful difference in our relative comparisons. Upon formation of hMSC-spheroids on D1, *GDF15* and *TGFB3* were upregulated by approximately 40-fold compared to the 2D control, whereas *BMP4* was downregulated by approximately 60-fold (Fig. 3c). As our dynamic 3D-MSC culture progressed to D7, *IL1B*, *BDNF*, and *BMP2* were upregulated by over 30-fold while *COL1A1* was downregulated by approximately 50-fold, compared to the early stage on D1 (Fig. 3d). The appreciable decrease in *COL1A1* from D1 to D7 suggested that the initial increase in ECM secretion only be necessary for structural organization of the 3D hMSC aggregates as described previously^23^. Comparisons of the 3D-MSC on D7 with 2D-MSCs showed that *IL1B* and *GDF15* were upregulated by approximately 40- and 90-fold, respectively. Particularly, *BMP2* was extensively upregulated by approximately 230-fold (Fig. 3e). Thus, the notable enhancement in differentiation potential towards chondrogenesis (reflected by *TGFB3* upregulation) and osteogenesis (indicated by *BMP2* upregulation) was observed in 3D-MSCs, which agreed with the results of previous studies^23,26^. Furthermore, other genes representing the communal attributes of hMSCs, mostly related to trophic factor secretion, were increased to some extent in our dynamic 3D-culture, which varies depending on the cell sources and 3D-culture conditions^23,26^.

### Significantly augmented production of MVs with dynamic 3D-hMSC culture

**Figure 4:**
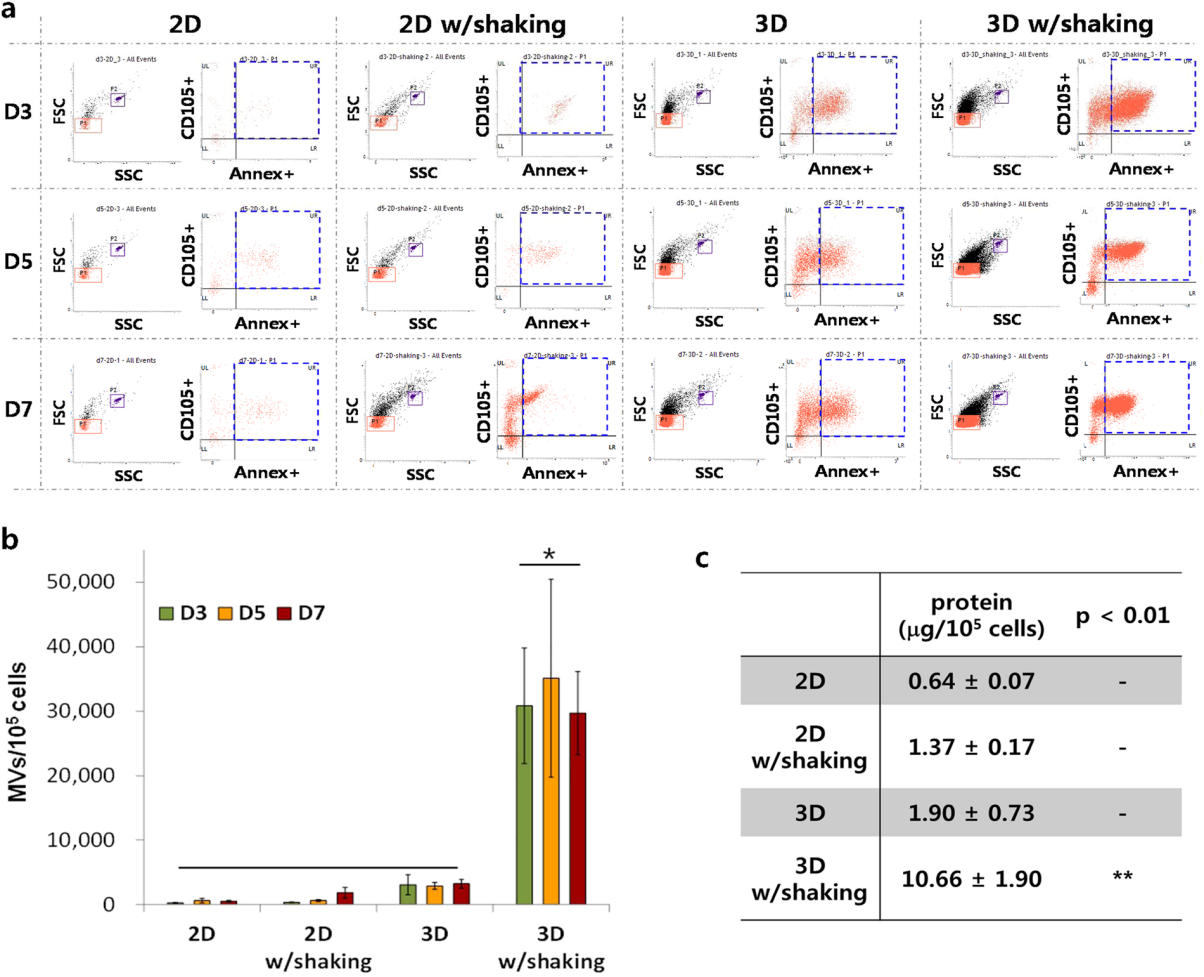
Significantly augmented production of MVs using dynamic 3D hMSC-culture. (**a**) A flow cytometric analysis for phenotyping and enumerating MVs collected from the groups of 2D, 2D w/shaking, 3D, and 3D w/shaking. Particles sized below 1.0 μm (red solid squares) were estimated using standard size beads, and those double-positive for anti-CD105 (hMSC surface marker) and anti-annexin V (lipid surface marker) were counted as hMSC-derived MVs on D3, D5, and D7 (blue dotted squares). Counting beads (purple solid squares) were used to calculate the absolute counts of MVs. (**b**) Quantitative comparison of counted MVs normalized to the cell numbers in corresponding culture groups. The highest enrichment of hMSC-derived MVs was observed in the 3D w/shaking group, which was approximately 100-fold greater than in the 2D control which contained only a few secreted MVs. Data are presented as the mean ± SEM. Differences among groups were evaluated by one-way ANOVA at a level of significance of p < 0.05. (**c**) A BCA protein quantification assay with samples collected on D7 which were normalized to the cell numbers of corresponding culture groups to compare all groups with the same standard. MVs collected from the 3D w/shaking group showed a significantly higher total protein concentration. Data are presented as the mean ± SEM. Differences among groups were evaluated by one-way ANOVA at a level of significance of p < 0.01 (**).

A previously established flow cytometric method^27,28^ was used for phenotyping and enumerating MVs in the groups of 2D, 2D w/shaking, 3D, and 3D w/shaking (Fig. 4a). Particles sized below 1.0 μm (red solid squares), estimated using standard size beads, and staining double-positive for anti-CD105 (hMSC surface marker) and anti-annexin V (lipid surface marker) were counted as hMSC-derived MVs on D3, D5, and D7 (blue dotted squares)^29^. Counting beads (purple solid squares) were used to calculate absolute counts of MVs, and the resulting counts of MVs were normalized to the cell numbers of corresponding culture groups (Fig. 4b). The highest enrichment of hMSC-derived MVs was found in the 3D w/shaking group, which was approximately 100-fold higher than in the 2D control containing only a few secreted MVs. The number of MVs collected from the 2D w/shaking group was generally higher, although not significantly, than that from the 2D group. Additionally, in culture without shaking, the formation of hMSC-spheroids (the 3D group) resulted in increased MV production, although the enrichment was not substantial, as observed in the 3D w/shaking group. Protein assays of samples on D7 normalized to the cell numbers of corresponding culture groups further supported these results, revealing significantly higher total protein concentrations in MVs in the 3D w/shaking group than in the other groups (Fig. 4c). In addition, the results obtained from using exosome-free FBS (Exo-free 3D-MVs) confirmed that the significantly reinforced MV production presented in this study was not influenced by particles originating from pre-filtered FBS (Supplementary Figure 3a).

### Morphology, size, and structure characterizations of MVs produced by dynamic 3D-hMSC culture

**Figure 5:**
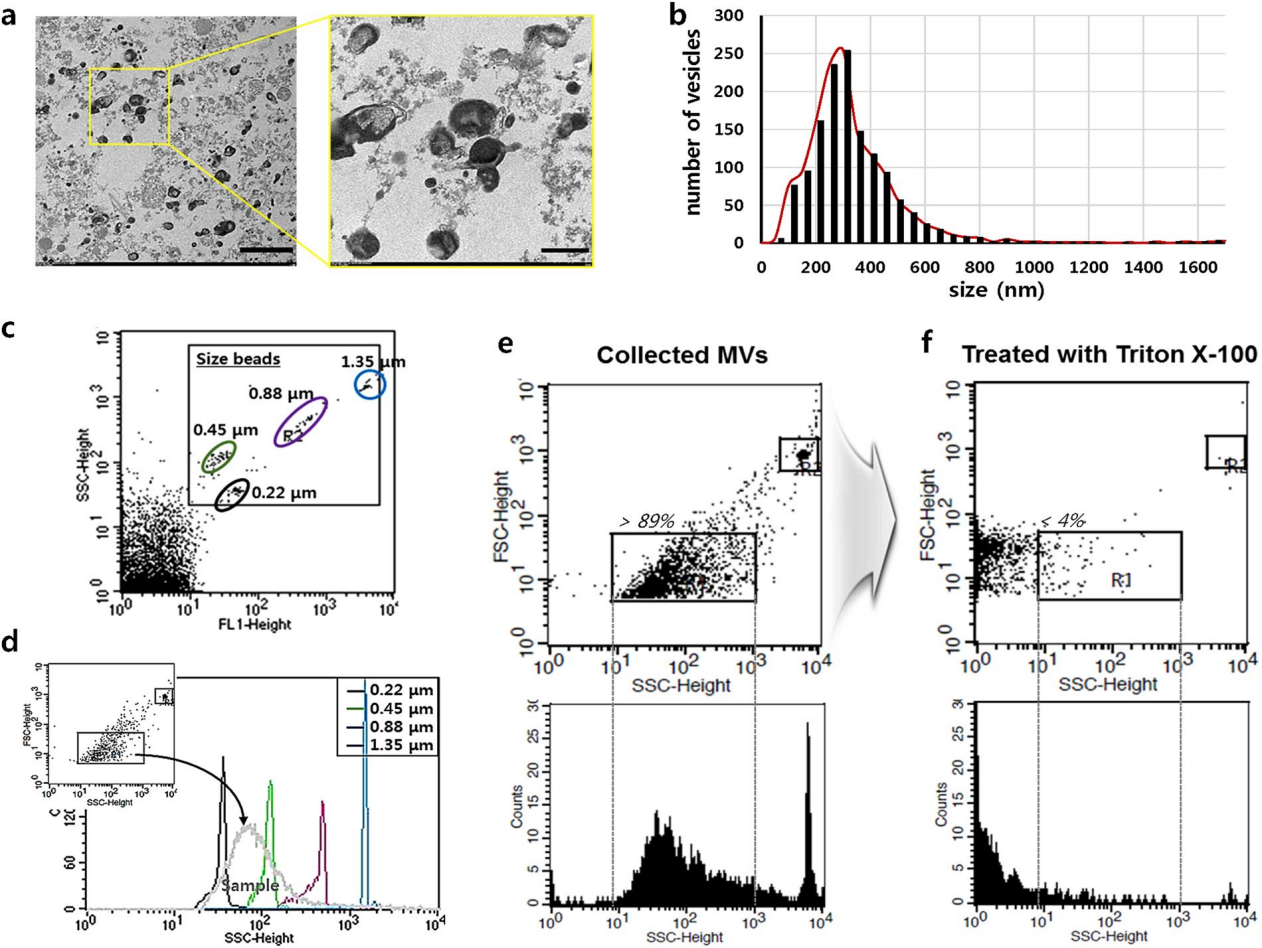
Morphology, size, and structure characterizations of MVs produced by dynamic 3D hMSC-culture. (**a**) TEM images of 3D-MVs. Most collected MVs displayed vesicular structures appearing rounded and bi-lipid layered, although differing in contrast and surface pattern. The size bars indicate 2,000 nm (left) and 500 nm (right). (**b**) Size distribution of the collected MVs obtained by measuring the sizes of individual vesicles on multiple TEM images (approximately 1,400 vesicles from 11 images). A peak in the MV diameter range was found at approximately 250–300 nm with over 80% of the collected MVs populated between 150 and 450 nm. (**c**) Particle size standards for flow cytometry obtained using the Nano Fluorescent Size Standard Kit. (**d**) A major population of the collected MVs was superimposed on the size calibration plot mainly in the range between 220 and 450 nm. (**e** and **f**) Flow cytometry results of the collected MVs before (**e**) and after (**f**) Triton-X 100 treatment, which indicated that the collected MVs were true lipid-membranous vesicles.

MVs collected from the 3D w/shaking group on D7 (named as 3D-MVs) were imaged by transmission electron microscopy (TEM). By analysing multiple TEM images, we visually characterized the morphology and size of collected MVs. Most MVs displayed vesicular structures, appearing rounded and bi-lipid layered, although with different contrast and surface pattern (Fig. 5a). Exo-free 3D-MVs also displayed similar morphologies (Supplementary Figure 3b). Size distribution of the collected MVs was obtained by measuring the sizes of individual vesicles on the TEM images (approximately 1,400 vesicles from 11 images) (Fig. 5b). A peak in the MV diameter range was observed at 250–300 nm; more than 80% of the collected MVs were populated between 150 and 450 nm, which was consistent with previous findings^30^. This result agrees with data obtained using the Nano Fluorescent Size Standard Kit which contains a size standard for microparticle flow cytometry (Fig. 5c). Superimposing the size calibration plot revealed that most of the collected MV population ranged in size from 220 to 450 nm (Fig. 5d). To confirm that the collected MVs were not protein aggregates but rather true vesicles enveloped by a lipid bi-layer, we examined their structural sensitivity with Triton X-100, which is commonly used for liposomal digestion^28,31^. Our flow cytometry results showed that most events gated by R1, where normal MVs were detected (Fig. 5e), disappeared (less than 4% remained) after Triton X-100 treatment (Fig. 5f), indicating that the collected MVs were liposome-like and Triton-sensitive vesicular structures. In addition, western blot analysis for markers of apoptotic bodies, such as thrombospondin (TSP) and C3b^32^, confirmed that few fragments of apoptotic bodies were contained in the collected MVs in this study (Supplementary Figure 4).

### Therapeutic inclusions in MVs produced by dynamic 3D-hMSC culture

The MVs mass-produced by our dynamic 3D-culture were analysed to validate their therapeutic properties. In addition to the dynamic 3D-hMSC culture employed in this study, we previously reported that the efficacy of hMSCs could be enhanced by pretreating the culture with ischemic brain extract (IBE)^33^. In our previous study, hMSCs pretreated with IBE were likely exposed to ischemic brain-mimicking microenvironments, thus resulting in significant enhancement of various trophic factor secretions related to neurogenesis and angiogenesis. Thus, we speculated that IBE pretreatment would also stimulate the secretion of MVs from hMSCs as a step in the process of their reinforced paracrine actions. As a result, a sufficient quantity of MVs was successfully obtained from IBE-pretreated hMSCs (named as IBE-MVs; Supplementary Figure 5), which were predicted to contain therapeutic compounds originating from the preconditioned hMSCs in this study.

**Figure 6:**
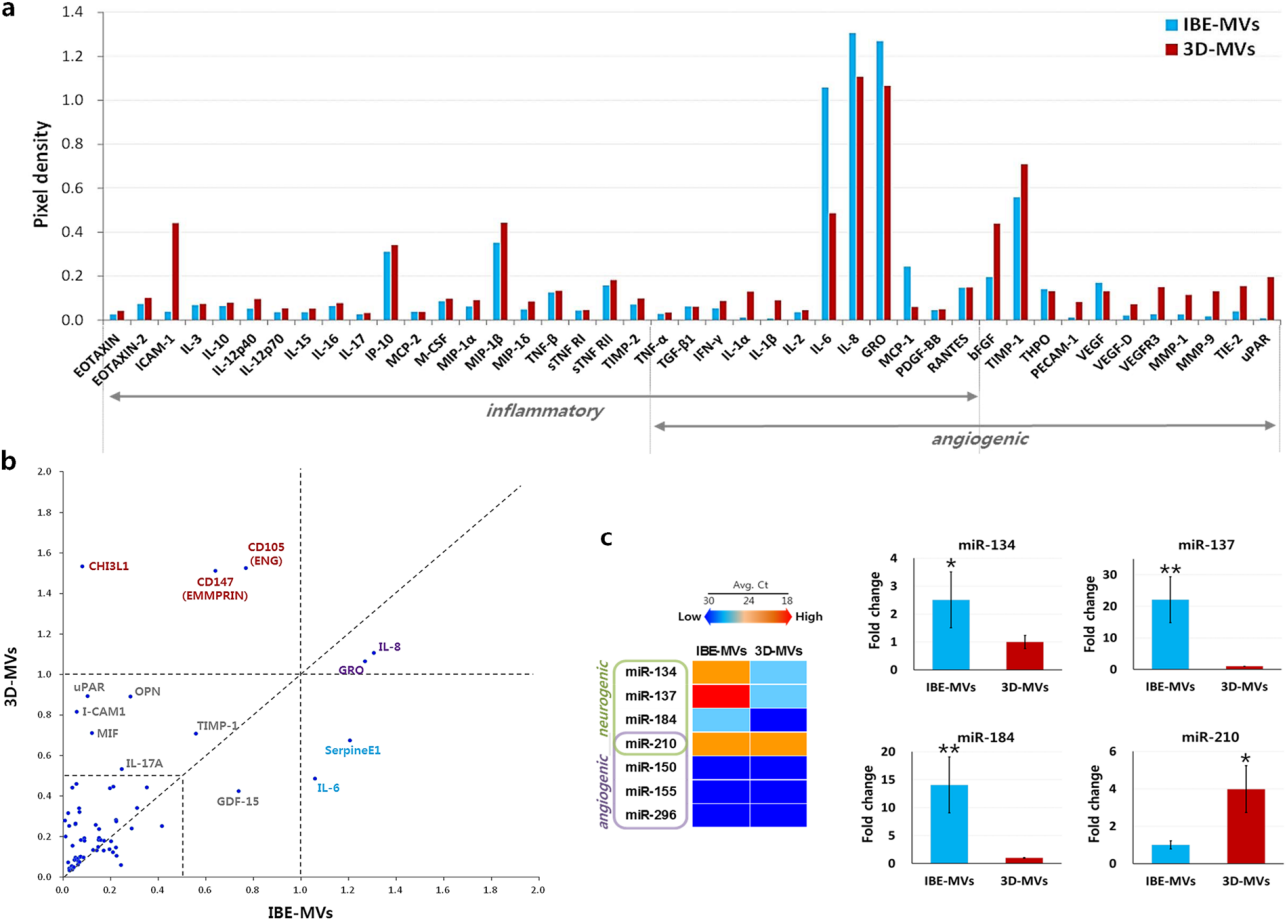
Therapeutic inclusions in MVs produced using dynamic 3D hMSC-culture. (**a** and **b**) Representative cytokines contained in both IBE-MVs and 3D-MVs were analysed using several cytokine array kits. IBE-MVs contained a variety of therapeutic cytokines related to immunomodulation and angiogenesis. In general, the cytokines detected in IBE-MVs were also found in 3D-MVs, but their amounts varied to some extent (**a**). Some cytokine inclusions were distinctive in the two groups, such that 3D-MVs appeared to contain large amounts of ICAM-1, bFGF, CHI3L1, CD147, and CD105, whereas considerable amounts of IL-6 and SerpineE1 were found in IBE-MVs (**b**). (**c**) qPCR assays for micro-RNAs included in MVs known as key players in neurogenic and/or angiogenic molecular signalling. IBE-MVs appeared to contain significantly higher levels of micro-RNAs related to neurogenesis such as miR-134, -137, and -184 compared to 3D-MVs. On the other hand, the level of miR-210 which is related to both neurogenesis and angiogenesis was significantly higher in 3D-MVs compared to in IBE-MVs. Regardless of the variations in some major compounds between the two groups, MVs collected from our 3D hMSC-bioprocess included high levels of various therapeutic cytokines and micro-RNAs related to immunomodulation, angiogenesis, and neurogenesis. Data are presented as the mean ± SEM. Differences among groups were evaluated by student’s t-test at a level of significance of p < 0.05 (*) or p < 0.01 (**).

Representative cytokines contained in both IBE-MVs and 3D-MVs were analysed using several cytokine array kits (Fig. 6a and b, and Supplementary Figure 6). As expected, IBE-MVs contained a variety of therapeutic cytokines related to immunomodulation and angiogenesis. In general, the cytokines detected in IBE-MVs were also found in 3D-MVs. Particularly, substantially high levels of IP-10, MIP-1β, IL-8, GRO, and TIMP-1 were found in both groups, but the included amounts showed some variation. Some inclusions of the cytokines were distinctive in the two groups, such that 3D-MVs appeared to contain large amounts of ICAM-1, bFGF, CHI3L1, CD147, and CD105, while considerable amounts of IL-6 and SerpineE1 were found in IBE-MVs (Fig. 6b). Additionally, qPCR assays for micro-RNAs were conducted to investigate the inclusion of representative key players for neurogenic and/or angiogenic molecular signalling within the MVs (Fig. 6c). The results showed that micro-RNAs related to neurogenesis such as miR-134, -137, and -184 were abundantly present in IBE-MVs^34,35^. However, the level of miR-210, which is related to both neurogenesis and angiogenesis^36^, was significantly higher in 3D-MVs compared to in IBE-MVs. Regardless of the variations in the major compounds between the two groups, MVs collected from our dynamic 3D-hMSC culture included abundant levels of various therapeutic cytokines and micro-RNAs related to immunomodulation, angiogenesis, and neurogenesis, comparable to those in IBE-MVs. Meanwhile, low levels of therapeutic inclusions were found in MVs originating from IBE- and 3D-fibroblasts (Supplementary Figure 6a and b).

The MVs collected from the 2D control (2D-MVs) and Exo-free 3D-MVs also contained various cytokines commonly observed in 3D-MVs (Supplementary Figure 7a). Interestingly, miR-210 was rarely expressed in the Exo-free 3D-MVs group, unlike in the 3D-MVs group (Supplementary Figure 7b). In contrast, no expression of the selected micro-RNA expression was detected in the 2D-MVs group.

### Angiogenic and neurogenic stimulation via MV supplementation

**Figure 7:**
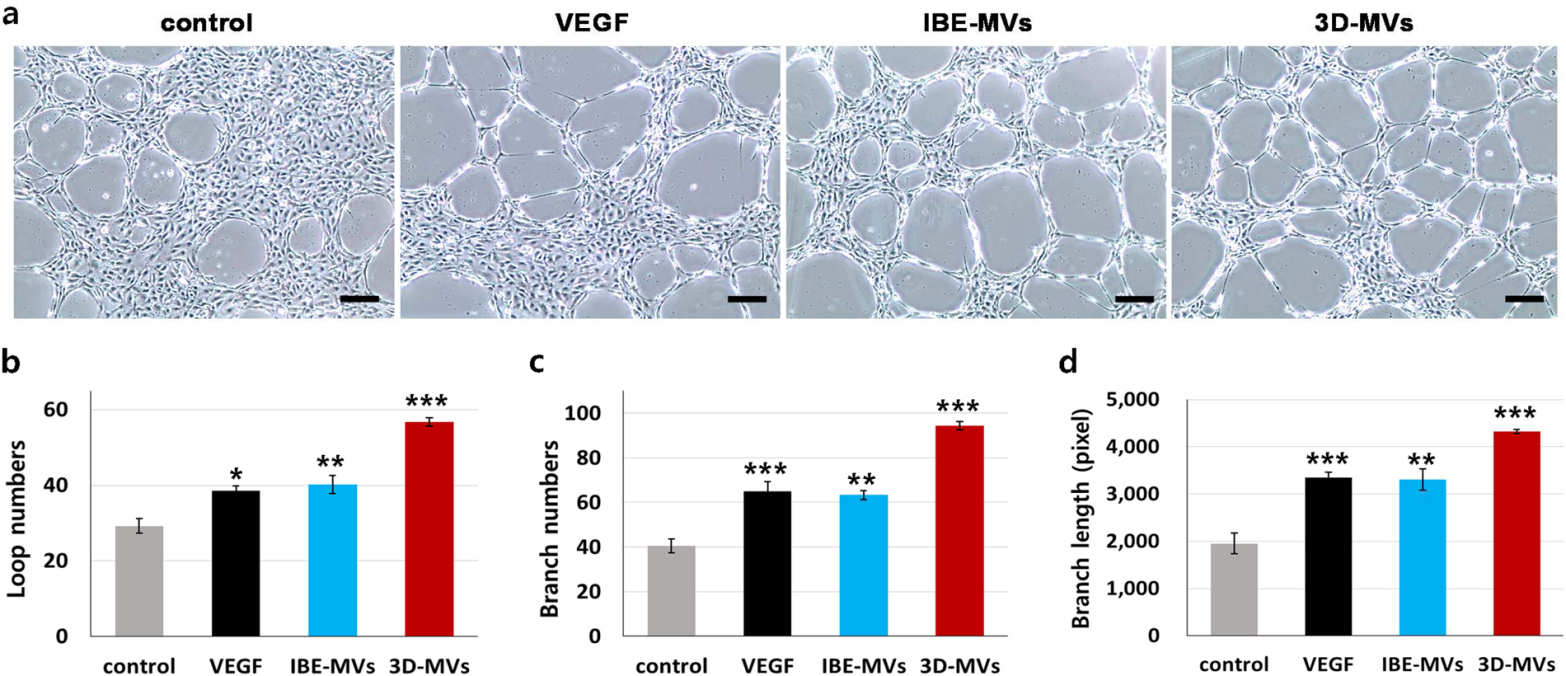
Angiogenic stimulation via MV supplementation. (**a**) The inducible capacity of MVs for vascular tube formation. 3 μg/mL of IBE-MVs and 3D-MVs were added to HUVECs plated on Matrigel, and resulting tube formation was observed along with a control (basal medium) and VEGF-treated groups by microscopy. The size bars indicate 200 μm. (**b**) Loop numbers (**c**) branch numbers and (**d**) branch length values of the resulting tube formations were quantitatively compared. While HUVECs treated with IBE-MVs showed similar levels to the VEGF-treated group, 3D-MVs demonstrated a greater capacity to stimulate HUVEC tube formation with higher significance than the other groups. Data are presented as the mean ± SEM. Differences among groups were evaluated by one-way ANOVA at a level of significance of p < 0.001 (***), 0.001 < p < 0.01 (**), or 0.01 < p < 0.05 (*).

We examined the therapeutic potency of collected MVs using *in vitro* models for angiogenesis and neurogenesis. To test the capacity to induce vascular tube formation, 3 μg/mL of IBE-MVs and 3D-MVs were introduced into human umbilical vein endothelial cells (HUVECs) plated on Matrigel (Fig. 7a), and the resulting tube formation was quantitatively compared with a control (basal medium) and vascular endothelial growth factor (VEGF)-treated groups in terms of loop numbers (Fig. 7b), branch numbers (Fig. 7c), and branch length values (Fig. 7d). VEGF supplementation, a conventional method for inducing tubular differentiation, triggered a significant increase in tube formation of HUVECs compared to the control group. While HUVECs treated with IBE-MVs showed similar levels as the VEGF-treated group, 3D-MVs demonstrated a greater capacity to stimulate HUVEC tube formation with an even higher significance compared to the other groups.

**Figure 8:**
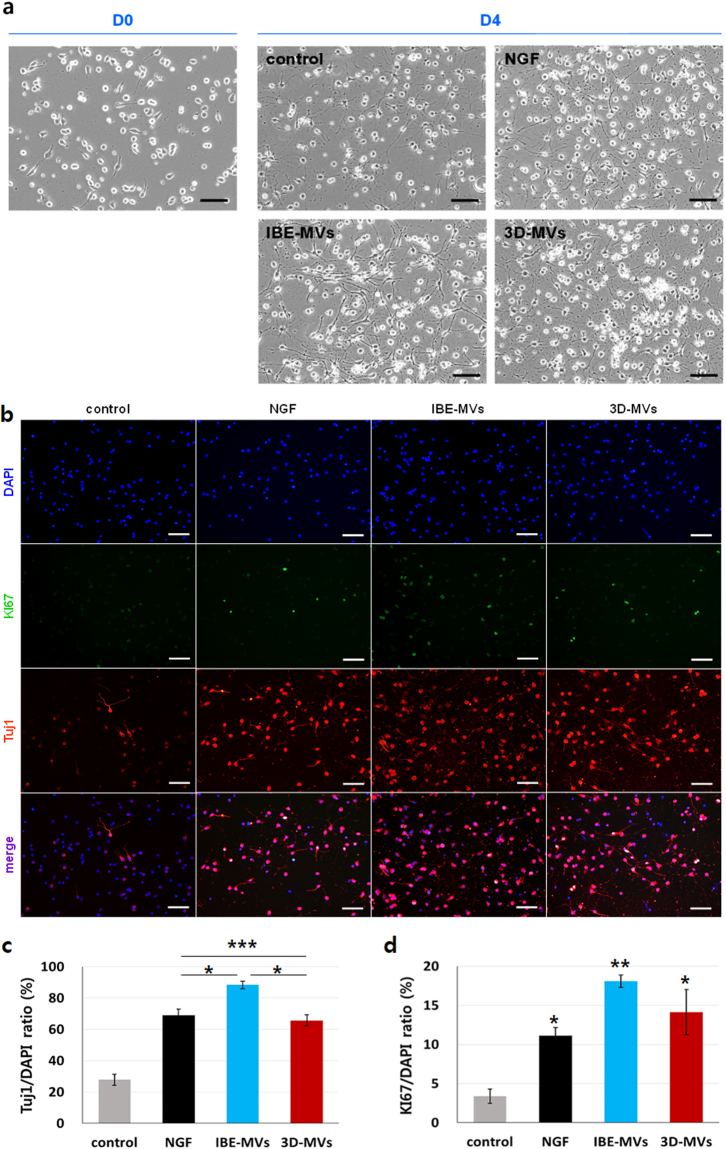
Neurogenic stimulation via MV supplementation. (**a**) Phase contrast images of the resulting neurogenic stimulation of MVs. 3 μg/mL of IBE-MVs and 3D-MVs were added to primarily cultured NSCs. Neural differentiation on D4 was compared with that in control (basal medium) and NGF-treated groups. The size bars indicate 100 μm. (**b**) Fluorescent image analyses of Tuj1 expression in NSCs along with Ki67 expression that denoted proliferating cells. The size bars indicate 100 μm. (**c**) Stimulated neurogenic differentiation of NSCs on D4 was quantified by counting cells positive for Tuj1 and normalized to DAPI-stained cells. IBE-MVs showed the highest capacity for stimulating neurogenic differentiation. 3D-MVs also induced neurogenic differentiation at a significantly higher level than the control as comparable to the NGF-treated group. (**d**) Proliferating NSCs on D4 were quantified by counting cells positive for Ki67 and normalized to DAPI-stained cells. IBE-MVs generally showed the greatest proliferation capacity, while 3D-MVs and NGF-treated groups were significantly higher than the control. Data are presented as the mean ± SEM. Differences among groups were evaluated by one-way ANOVA at a level of significance of p < 0.001 (***), 0.001 < p < 0.01 (**), or 0.01 < p < 0.05 (*).

To examine the capacity for stimulating neurogenesis, we added 3 μg/mL of IBE-MVs and 3D-MVs to primarily cultured neural stem cells (NSCs) (Fig. 8a). The resulting neural differentiation was evaluated on D4 by quantifying the Tuj1 expression in NSCs compared to in a control (basal medium) and nerve growth factor (NGF)-treated groups (Fig. 8b and c). Proliferating NSCs were also quantified simultaneously by counting cells positive for Ki67 (Fig. 8b and d). IBE-MVs showed the highest capacity to stimulate the neural differentiation of NSCs with a greater proliferation capacity compared to the other groups. However, 3D-MVs also induced neural differentiation of NSCs comparable to that by the NGF-treated group and retained their proliferation up to D4 at a significantly higher level than the control

Similar therapeutic potency was observed for the Exo-free 3D-MVs group, as both HUVEC tube formation and NSC neurogenic differentiation were significantly stimulated. Additionally, the 2D-MVs group showed relatively lower degrees of efficacies compared to the Exo-free 3D-MVs group with levels comparable to the conventional positive controls of VEGF and NGF supplementation (Supplementary Figures 8 and 9).

## Discussion

Extracellular vesicles (EVs) released by cells play a crucial role in regulating numerous physiological events and orchestrating interactions between cells and the surrounding microenvironments^30^. In addition to artefacts or fragments of degenerated or dead cells, such as apoptotic bodies, two classes of EVs are considered the main source of intercellular communication between living cells, exosomes and MVs, which are distinguished by their biogenic mechanisms^30,37^. Exosomes are released by the exocytosis of multivesicular bodies existing as intraluminal vesicles in cells, typically with sizes of 40–100 nm. MVs are released into the extracellular space by budding directly from the plasma membrane, and thus are relatively larger than exosomes, with sizes between 100 and 1,000 nm^30,38,39^. The two classes of EVs are reported to be largely analogous in the resulting biological functions and contain multiple cytosolic proteins and nucleic acids relevant to a variety of biological phenomena in the surrounding and/or remote regions^37^. While the practical advantages and disadvantages of their therapeutic applications are still in discussion, recent studies have suggested that MVs contain greater amounts of cargo compounds, proteins, and/or RNAs, as well as larger amounts of segregated plasma membrane domains than exosomes. This may increase the possibility of fusion with a target cell that initiates a specific molecular signalling pathway and invokes phenotypic changes^37,40^. Therefore, in the present study, MVs derived from hMSCs were investigated and various therapeutic aspects of EVs are discussed, regardless of the classification.

MSCs are a potentially rich source of a variety of therapeutic factors that inhibit cell apoptosis, stimulate proliferation, and promote vascularization of affected tissues and are capable of immunomodulation^41^. In turn, the importance of EVs released by the MSCs was further asserted as they were thought to play major roles in the paracrine functions, to which most of the positive outcomes of MSC therapies are attributed^7–9^. MSC-derived EVs are characterized as nano-sized membranous vesicles containing various therapeutic cytokines and RNAs, which may possess therapeutic potency similar to that of whole MSCs^42^. Moreover, MSC-derived EVs, similar to whole MSCs, have the advantage of homing ability as bearing various MSC membrane antigens that have a binding affinity to ligands on specific cell membranes or the ECM in the diseased sites^43–45^. In addition, the biological activities of EVs are mainly enzyme-driven, and thus their therapeutic functions can be inherently regulated by being activated or attenuated in accordance with the severity of the disease-precipitating microenvironment^46^. As many recent studies have demonstrated the outstanding therapeutic advantages of MSC-derived EVs over MSC infusion therapy in a series of animal models, MSC-derived EVs may provide a novel therapeutic modality^42,47^. Large-scale production is critical for the clinical translation of this promising strategy, along with the requirements for regulatory approval such as understanding EV biology and safety, etc^15,42,48,49^. Some studies have reported that immortalization of MSCs enables sustainable production of MSC-derived EVs without influencing their therapeutic efficacy^48^, although safety concerns have been raised for the procedures^42^. However, this approach used conventional monolayer culture methods that do not accurately reflect *in situ* microenvironments where MSCs extensively release paracrine factors including EVs, requiring the culturing of numerous batches of MSCs. Thus, costly and labour- and time-consuming procedures are required to obtain clinically feasible amounts of EVs.

As a model system to understand the *in vivo* scenarios in tumour biology or embryonic development, cellular aggregates have long been studied to mimic physiologically relevant microenvironments^50,51^. MSCs were also found to spontaneously form cellular aggregates in suspension culture, which appeared to mimic the mesenchymal condensation events in developmental stages^18,52^. Numerous recent studies have suggested that these 3D MSC-aggregates have advantages over monolayer cultures in several therapeutic applications^23,47^. For example, 3D-cultured MSC-aggregates significantly augmented various therapeutic biomolecular secretions including anti-inflammatory, proangiogenic, and promitotic factors^19,23,24,53,54^. In addition, replated MSC-aggregates displayed enhanced differentiation efficiency toward adipogenic, chondro-osteogenic, and potentially epithelial-like or neuronal-like phenotypes, suggesting that this 3D MSC-culture technique can be used as a preconditioning strategy to reinforce the therapeutic properties of MSCs^23,53,54^. Therefore, in this study, we hypothesized that the production of therapeutic MVs could be amplified by enhancing the biological functions of MSC-aggregates as dynamic 3D-culture progressed. First, to achieve the large-scale formation of size-controlled hMSC-spheroids in a reproducible manner, we adopted and modified a PEG hydrogel microwell array platform with an inverted-pyramidal opening structurally adjoining each cylindrical microwell^22^. The unique features of our modified PEG microwell array completely prevented cell loss during the large-scale formation of hMSC-spheroids, resulting in significant cost-saving through reduced wastes of expensive cell materials. We also achieved highly reproducible and precisely controlled sizes and cell numbers of hMSC-spheroids. Subsequently, a dynamic culture condition was applied to the microwell array containing 1,225 hMSC-spheroids, which resulted in an approximately 100-fold increase in hMSC-derived MV production compared to in the 2D control. Comprehensive characterization of the MVs collected from our dynamic 3D hMSC-culture confirmed their features as nano-sized and lipid-membranous vesicles, in agreement with other studies, which were distinct from whole MSCs as well as small biomolecules^30^.

In addition to the dynamic 3D MSC-culture method presented in this study, we previously reported that pretreatment with IBE in the hMSC-culture significantly improved the efficacy of hMSCs in ischemic stroke by increasing the secretion of relevant trophic factors^33^. We predicted that such reinforced paracrine actions of MSCs by both culture methods would mainly involve a variety of MV-mediated biomolecular signals. In addition to achieving mass-production of MVs, our results demonstrated that both IBE-MVs and 3D-MVs contained abundant levels of various cytokines related to the inflammatory and angiogenic properties of hMSCs, although the levels of some major compounds varied in both groups. Furthermore, previous studies suggested that the therapeutic functions of EVs may not be attributable only to the cytokine inclusions, as EVs also contain a range of functional RNAs including micro-RNAs, which are known as key players in various physiological events^55,56^. While examining a set of micro-RNAs, we found that the micro-RNAs contained in IBE-MVs generally showed stronger associations with neurogenesis, as expected from our previous study^33^. This result suggests that MSCs pre-educated in a disease-specific microenvironment produce MVs with the desired internal compositions for a targeted disease. However, the IBE-MVs were only used for comparisons with our 3D-MVs in this study, as using IBE in the hMSC culture cannot be conducted clinically because of difficulties in acquiring brain biopsies from patients as well as safety concerns from numerous unidentified factors included in the tissue extracts. The therapeutic potencies of IBE-MVs and 3D-MVs were also demonstrated in this study; supplementations of IBE-MVs and 3D-MVs resulted in comparable or significantly higher efficacies in HUVEC tube formation and NSC proliferation/differentiation compared to the conventional positive controls of VEGF and NGF supplementation, respectively. We confirmed that these results were only minimally influenced by possible EV contaminants from the pre-filtered FBS used in this study. Unlike in the 3D-MVs group, miR-210 was not highly expressed in the Exo-free 3D-MVs group, while supplementation of Exo-free 3D-MVs resulted in significantly stimulated angiogenesis and neurogenesis. This result suggests that the miR-210 content in the 3D-MVs group originate from the EVs initially contained in FBS, albeit more detailed investigations are necessary to confirm this. In contrast, expression of the selected micro-RNAs was not detected in the 2D-MVs group, while various therapeutic cytokines were found to be included. This may be related to their relatively lower efficacy in angiogenic and neurogenic stimulation compared to in the Exo-free 3D-MVs group. However, our study provides limited mechanistic evidence to rationally explain which composition between cytokines and micro-RNAs in the MVs played a more significant role in neurogenic and/or angiogenic stimulation. Although the information on the biomolecular compositions of the MVs allowed for a rough prediction of their therapeutic capabilities, the precise mechanisms rendering the 3D-MVs more effective in the angiogenesis model and IBE-MVs in the stimulation of NSC proliferation/differentiation remain unknown. Further ongoing studies in our laboratory may improve the understanding of the multiple key factors that predominantly direct MV-mediated therapeutic outcomes as well as clarify their exact roles in therapeutic mechanisms, providing information needed to custom-engineer therapeutic properties of MVs initially primed for a targeted disease.

Additionally, high-throughput gene expression analysis carried out using a PCR array kit for hMSCs revealed that our hMSC-spheroids retained their stemness and hMSC marker gene expression during dynamic 3D-culture as compared to the 2D control, while the gene expressions indicating hMSC functions such as differentiation potential and trophic factor secretion were generally enhanced as shown in many previous reports^23,26^. This gene expression profile data, although preliminary, may provide crucial information for evaluating the generic status of our cell material. To reproducibly and reliably produce MVs as therapeutic agents, all procedural parameters must be controlled. Particularly, as the most important starting material, hMSC-spheroids should retain their characteristics within defined and universally accepted standards for hMSCs^57^. Characteristics of the 3D-cultured hMSCs may be governed by several key culture parameters such as cell source, cell density of spheroids, culture medium, and culture period, etc. To reduce unknown discrepancies in the biological and/or physical properties of the resulting MVs, detailed characterization data of hMSC-spheroids with varying culture conditions will be collected in our future studies, which would contribute to minimizing the inconsistent therapeutic effects of MVs in diverse applications.

MSC-derived MVs are considered a highly attractive therapeutic strategy, as a safe, effective cell-free treatment can circumvent the current limitations of MSC and/or soluble biomolecule infusion therapies. They are currently under active investigation to address a range of complexities in the molecular mechanisms of MV biogenesis and therapeutic functions, standardized protocols to isolate and purify the small vesicles with well-defined classification, and desired recruitment of the multifaceted proteomic and genomic compositions in the MVs, etc. Thus far, however, few studies have endeavoured to alleviate concerns about the affordability for the large-scale production of MSC-derived MVs. In this study, we presented a simple and effective 3D-bioprocessing method for the scalable production of therapeutic MVs from hMSCs. Our method, which is also applicable to other cell types and adjustable for different MSC-preconditioning approaches, may be useful for other research purposes or clinical/commercial uses, which require the large-scale production of EVs.

## Methods

### Fabrication of PEG hydrogel cylindrical microwell arrays with inverted-pyramidal openings

To customize the PEG hydrogel cylindrical microwell arrays to include inverted-pyramidal openings, two moulding processes were used, the polydimethylsiloxane (PDMS, SYLGARD® 184 SILICONE ELASTOMER, Dow Corning, Midland, MI, USA) moulding process to form a PDMS counter-structured mould and PEG moulding process to achieve the final PEG hydrogel microwell arrays. The first moulding process required a silicon (Si) master mould, which had the same structure as the final microwell design, and was fabricated on a Si wafer (Waferbiz, Seoul, Korea) using conventional micro-fabrication technologies such as Si wet etching for the inverted-pyramidal openings, dry etching for the cylindrical structures, and sputtering and photolithography processes for the individual wet etching and dry etching masks. The fabricated Si master mould was used for the first moulding process to form a PDMS counter mould, which had an inversed Si master mould shape. A PDMS solution (composition ratio of resin to curing agent of 10 to 1) was poured onto the Si master mould, and cured at 95 °C for 2 hours. The PDMS structures, detached from the Si master mould, were used for the second PEG moulding process to form the final PEG hydrogel microwell arrays. For the second PEG moulding process, a PEG solution which consisted of phosphate buffered saline (PBS, Lonza, Basel, Switzerland), photoinitiator (BASF, Ludwigshafen, Germany) and poly(ethylene glycol)1000 dimethacrylate (PEGDMA 1000, Polysciences, Inc., Warrington, PA, USA) in the ratio of 10:0.1:1 respectively, was poured on a slide glass pre-coated with 3-(trimethoxysilyl)propyl methacrylate (TMSPMA. Sigma, St. Louis, MO, USA). The PEG solution was stamped with the PDMS counter mould, and UV-induced PEG crosslinking was carried out at 7 W/cm^2^ for 65 sec (Omnicure^®^ S2000, Excelitas Technologies Corp., Waltham, MA, USA). After the PDMS counter mould was removed, the fabricated PEG hydrogel microwell arrays were stored in 70% ethanol solution until use.

### 3D-culture of hMSCs using microwell arrays

hMSCs (PT2501, Lonza, Basel, Switzerland) were cultured in a 5% CO_2_ incubator at 37 °C. The growth medium was low-glucose Dulbecco’s modified Eagle’s medium (DMEM, Gibco, Grand Island, NY, USA) containing 10% foetal bovine serum (FBS, Hyclone, Logan, UT, USA) or exosome-free FBS (System Biosciences, Palo Alto, CA, USA) and 1% antibiotics-antimycotics (Gibco). The FBS supplemented in our hMSC culture medium was pre-filtered through 0.22-μm membranes to eliminate most of the FBS-originated MVs in the analysis. To seed hMSCs into the microwell arrays, the cells were trypsinized using TrypLE Express (Gibco) and counted with a haemocytometer. These were then suspended in 200 μL of culture medium at a density of 5 × 10^5^ cells/array, and drop-seeded into the microwell arrays. The hMSCs coalesced within the microwells and agglomerated as spheroids were observed by phase-contrast microscopy at 20 min and 1 day after cell seeding, respectively. They were subsequently cultured at 30-rpm in an orbital-shaker for 7 days. Mouse fibroblasts (NIH3T3, ATCC, Manassas, VA, USA) were also cultured in high-glucose DMEM (Gibco) containing 10% FBS (Hyclone) and 1% antibiotics-antimycotics (Gibco) and used to compare the cytokine inclusions in the collected MVs with those from hMSCs.

### Isolation of MVs from hMSC-culture medium

MVs were isolated from the collected media by sequential centrifugation at 2,500 × *g* for 10 min to remove the cell debris/apoptotic bodies and at 14,000 × *g* for 45 min at 10 °C to obtain the MV pellets^58,59^.

### Live and dead assay of hMSC-spheroids

Cellular viability of the hMSC-spheroids was assessed using a LIVE/DEAD Viability/Cytotoxicity Kit (Invitrogen, Carlsbad, CA, USA). The hMSC-spheroids were formed as described above and collected on D3, D5, and D7 in the 3D w/shaking group. The images of calcein AM (live cells) and EthD-1 (dead cells) were obtained using a fluorescence microscope (EVOS, Advanced Microscopy Group, Bothell, WA, USA).

### Histological analysis of hMSC-spheroids

The hMSC-spheroids were fixed using 4% (w/v) paraformaldehyde (Sigma). After washing with PBS, the fixed samples were placed in an egg albumin solution diluted in glycerol and then centrifuged at 3,000 rpm for 5 min, followed by a conventional paraffin-embedding protocol. The sections were immersed in xylene (Junsei, Tokyo, Japan) for deparaffinization and then placed in decreasing concentrations of ethanol for rehydration. Samples were stained with H&E (Sigma) and M&T (Sigma) using conventional protocols, and observed under a microscope (EVOS, Advanced Microscopy Group).

### Growth kinetics analysis

Cell numbers were quantified using a DNA quantification assay kit (CyQUANT NF Cell Proliferation Assay Kit, Invitrogen) following the manufacturer’s instructions.

### qPCR array for hMSC-spheroid characterization

Total RNAs from hMSCs were extracted using Trizol reagent (Invitrogen) according to the manufacturer’s instructions. The 84 genes representing characteristics of hMSCs were quantitatively analysed using a commercial qPCR array kit (RT^2^ Profiler^TM^ PCR array: Human Mesenchymal Stem Cells, Qiagen, Hilden, Germany) following the manufacturer’s instructions.

### Flow cytometric quantification and size characterization of MVs

A flow cytometric method was used for phenotyping and enumerating the MVs, as previously reported^27,28^. Isolated MVs were double-stained with anti-CD105 (AbD Serotec, Kidlington, UK) and anti-annexin V (BD Pharmingen, San Jose, CA, USA)^29^ to confirm that the counted MVs originated from hMSCs. The absolute counts of MVs were analysed using both forward scatter (FSC) and side scatter (SSC) in logarithmic mode (FACS Verse flow cytometer and BD FACSuiteTM software, BD Biosciences, San Jose, CA, USA). Standard beads of 0.22, 0.45, 0.88, and 1.35 μm (Nano Fluorescent Size Standard, Spherotech, Lake Forest, IL, USA) were used to estimate the size of counted MVs; in particular, the position of the gate R1 was preliminarily determined by using standard beads of 1.35 μm to detect particles smaller than 1 μm. Counting beads (7 μm, CountBrightTM Absolute Counting Beads, Thermo Fisher, Waltham, MA, USA) were used and gated at R2 to calculate the absolute counts of MVs by following the manufacturer’s instructions (1):

$$\frac{{\rm{MVs}}}{{\rm{\mu }}{\rm{l}}}=\,(\frac{total\,events}{assigned\,bead\,count})\,\times \,(\frac{standard\,bead\,event}{volume\,of\,sample})$$

The counted MVs were normalized by cell numbers of the corresponding culture group so that the rates of MV production were commensurate among the groups with the same standard.

To confirm that the collected MVs were not protein aggregates but rather true vesicles enveloped by a lipid bi-layer, we examined their structural sensitivity using Triton X-100, which is commonly used for liposomal digestion^28,31^. The collected MVs were immersed in 3% Triton X-100 (Sigma) solution diluted in PBS and measured using a flow cytometer as described above.

### Protein quantification of MVs

The protein contents of MVs were measured by using the Micro BCA Protein Assay Kit (Thermo Scientific) according to the manufacturer’s instructions. The measured protein quantity was normalized to the cell numbers of the corresponding culture group to compare all groups with the same standard.

### Morphology and size characterization of MVs using TEM

MVs were fixed overnight with 2.5% (w/v) glutaraldehyde (Sigma) in 4% (w/v) paraformaldehyde solution (Sigma) at 4 °C. After incubation in 1% (w/v) OsO_4_ for 1 hour, the samples were dehydrated in a series of ethanol diluents, passed through propylene oxide, and embedded in epoxy resin (Epok 812, 02–1001, Oken, Japan). Ultrathin sections (60 nm) were collected on 200-mesh nickel grids and stained for 10 min in 1% uranyl acetate and Reynolds’ lead citrate. The samples were observed with a Hitachi HT7700 electron microscope (Tokyo, Japan) at 80 kV. Approximately 1,400 MVs were randomly selected from eleven TEM images and their sizes were measured using ImageJ software (NIH, Bethesda, MD, USA) for particle size distribution analysis.

### Western blotting analysis for apoptotic bodies

Lysates of MVs, cell debris, and whole hMSCs for western blotting were extracted with RIPA buffer containing protease inhibitors and phosphatase inhibitors (Roche, Basel, Switzerland). Protein concentrations were determined by the Bradford assay. Lysates (25 μg) were electrophoresed on SDS-polyacrylamide gels and transferred to PVDF membranes (Millipore, Billerica, MA, USA). After blocking, the membranes were incubated overnight at 4 °C with primary antibodies: human anti-thrombospondin (diluted at 1:500, BD Pharmingen) and human anti-C3b (diluted at 1:500, BD Pharmingen). The membranes were then incubated with secondary antibodies (diluted at 1:5000, Cell Signaling Technology, Danvers, MA, USA). Bands were visualized with enhanced chemiluminescence (Millipore).

### Preparation and treatment of IBE

All animal experiments were approved by the Institutional Animal Care and Use Committee (IACUC) of Samsung Biomedical Research Institute (SBRI, Approval No. 20160106001) and performed according to the Institute of Laboratory Animal Resources (ILAR) guidelines. All animals were maintained in compliance with the relevant laws and institutional guidelines of Laboratory Animal Research Centre (LARC; AAALAC International approved facility, No. 001003) at the Samsung Medical Centre. Anaesthesia was induced using a face mask in male Sprague-Dawley (SD) rats (7–8 weeks old, 250–300 g) with 4% isoflurane (Hana Pharm, Gyeonggi-do, Korea) and maintained with 1.5% isoflurane in 70% N_2_O and 30% O_2_. The body temperature was maintained at 37.0–37.5 °C (measured rectally) with heating pads. We induced a transient middle cerebral artery occlusion (tMCAo) using a previously described intraluminal vascular occlusion method modified in our laboratory^60^. The IBE was obtained 3 days after a 90-min tMCAo. The ipsilateral hemispheres were homogenized in DMEM (150 mg/mL) on ice. After centrifugation at 10,000 × g at 4 °C for 10 min, the supernatants were collected and stored at −70 °C. hMSCs were seeded in T75 flasks at a density of 6 × 10^5^ cells per flask and cultured at 37 °C in 5% CO_2_ for 24 hours. Stored IBE was thawed and centrifuged at 2,500 × *g* for 10 min to eliminate the debris. After a 5-fold dilution with DMEM, IBE was centrifuged at 14,000 × *g* for 45 min at 10 °C and filtered with a 0.2-μm bottle top filter to remove MVs from the tissue extracts. hMSCs were exposed to the prepared IBE for 24 hours.

### Analyses of therapeutic inclusions contained in MVs: cytokines and micro-RNAs

High-throughput screening of a variety of cytokine inclusions in MVs associated with therapeutic capacity was carried out using several cytokine array kits, such as the Proteome Profiler^TM^ Human XL Cytokine Array Kit (R&D Systems, Minneapolis, MN, USA), Human Angiogenesis Antibody Array (Abcam, Cambridge, UK), and Human Inflammation Antibody Array (Abcam), following the manufacturer’s instructions. To determine the key players in neurogenic and/or angiogenic molecular signalling in the MVs, we performed micro-RNA expression analysis using qPCR assays. Total RNAs of the IBE- and 3D-MVs were extracted using Trizol reagent (Invitrogen) according to the manufacturer’s instructions. Expression levels of the micro-RNAs were measured using stem-loop RT primers and TaqMan PCR Mastermix (Applied Biosystems, Foster City, CA, USA) and were normalized against the levels of miR-16. The primers for the micro-RNAs were included in TaqMan® MicroRNA Assays (Applied Biosystems), miR-16 (Cat. No. 4427975–000391), miR-134 (Cat. No. 4427975-001186), miR-137 (Cat. No. 4427975-001129), miR-184(Cat. No. 4427975-000485), miR-210 (Cat. No. 4427975-000512), miR-150 (Cat. No. 4427975-462465), miR-155 (Cat. No. 4427975-002623), and miR-296 (Cat. No. 4427975-002101). Relative comparisons of micro-RNA expression levels between the IBE- and 3D-MVs were calculated using the comparative CT method (2^−ΔΔCT^).

### HUVEC culture and tube formation assay

HUVECs were purchased from ATCC and cultured on 1% gelatin-coated flasks in M199 supplemented with 20% FBS, 5 U/mL heparin (Sigma), 3 ng/mL bFGF (Invitrogen), and antibiotics-antimycotics (Gibco). Passage 3–5 HUVECs, were seeded on a Matrigel-coated μL-slide in M199 supplemented with 1% FBS, 5 U/mL heparin, and antibiotics-antimycotics. Simultaneously, HUVECs were treated with 3 μg/mL of IBE- or 3D-MVs without growth factor supplements, and compared with a control (basal medium) and VEGF-treated groups (100 ng/mL, Life Technologies, Carlsbad, CA, USA). Tube formation of the HUVECs was observed 6 hours after treatment and loop numbers, branch numbers, and branch lengths were quantified using ImageJ software.

### Primary culture of rat NSCs

SD rat embryos were obtained at 14.5 days. The cerebral cortex was harvested and immersed in DMEM/F12 (Gibco). The meninges were removed and washed in DMEM/F12 by centrifugation for 5 min at 500 × g. The pellet of the cerebral cortex was treated with Accutase (Biowest, Nuaillé, France). The Accutase digest was neutralized by adding DMEM/F12 containing 1% N2 supplements, 20 ng/mL EGF, 20 ng/mL FGF, and 1% antibiotics-antimycotics. Cells were then spread by pipetting up and down several times and pelleted by centrifugation. After re-suspension in the culture medium, the cells were transferred into T25 flasks and cultured in a 37 °C incubator. After 5–7 days of culture, the neurospheres were passaged to expand the NSCs for future experiments.

### Analysis of neurogenic differentiation of NSCs

NSCs were plated at 2.5 × 10^5^ cells/mL on 24 well plates coated with 20 μg/mL of poly-D-lysine (Sigma) using DMEM/F12 supplemented with N2 (Gibco), 20 ng/mL bFGF (Invitrogen), 20 ng/mL EGF (Invitrogen), and antibiotics-antimycotics (Gibco). For analysis, the NSCs were treated with a 3 μg/mL of IBE- or 3D-MVs without growth factor supplements. These experimental groups were compared with a control (basal medium) and NGF-treated (100 ng/mL, Thermo Scientific) groups. After 4 days of culture, neurogenic differentiation of the NSCs was analysed using a conventional immunocytochemistry protocol for rabbit anti-Ki 67 (diluted 1:50, Abcam) and mouse anti-Tuj1 (diluted 1:100, Millipore). Secondary antibodies were sequentially applied as follows: DyLight-labeled anti-rabbit IgG (diluted 1:200, 594 nm, Abcam) and DyLight-labeled anti-mouse IgG (diluted 1:200, 488 nm, Vector Laboratories, Burlingame, CA, USA). After mounting using Vectashield^TM^ with 1.5 μg/mL 4′-6′ diamidino-2-phenylindole (DAPI) (Vector Laboratories), samples were imaged using a fluorescence microscope (EVOS, Advanced Microscopy Group), and positively stained NSCs were quantified using ImageJ software.

### Statistical analysis

Quantitative analyses were conducted on more than three independent experiments (n ≥ 3), and data were presented as the mean ± standard error of the mean (SEM). Differences among groups were evaluated by one-way analysis of variance (ANOVA, Tukey’s post-hoc test) at a level of significance of p < 0.001 (***), 0.001 < p < 0.01 (**), or 0.01 < p < 0.05 (*) (SPSS version 20, SPSS Inc., Chicago, IL, USA).

### Data availability

All data generated or analysed in this study are included in this published article (and its Supplementary Information files).

## Electronic supplementary material

Supplementary figures
