# Supplementary material for: Efficient scalable production of therapeutic microvesicles derived from human mesenchymal stem cells
Source: Sci Rep. 2018 Jan 19;8:1171. doi: 10.1038/s41598-018-19211-6 (PMC5775399; doi:10.1038/s41598-018-19211-6)
Supplement: Supplementary file 1 — Supplementary figures [file 41598_2018_19211_MOESM1_ESM.pdf]

Efficient scalable production of therapeutic microvesicles derived from human mesenchymal stem cells

Jae Min Cha[1], Eun Kyoung Shin[2], Ji Hee Sung[2], Gyeong Joon Moon[2,3], Eun Hee Kim[2,3], Yeon Hee Cho[2], Hyung Dal Park[4,5], Hojae Bae[6], Jinseok Kim[4]*, Oh Young Bang[2,3,7]*

[1] Medical Device Research Centre, Research Institute for Future Medicine, Samsung Medical Centre, Seoul 06351, Republic of Korea

[2] Translational and Stem Cell Research Laboratory on Stroke, Samsung Medical Centre, Seoul 06351, Republic of Korea

[3] Stem Cell and Regenerative Medicine Centre, Research Institute for Future Medicine, Samsung Medical Centre, Seoul 06351, Republic of Korea

[4] Centre for Bionics of Biomedical Research Institute, Korea Institute of Science and Technology, Seoul 03722, Republic of Korea

[5] Department of Mechanical Engineering, Yonsei University, Seoul 02792, Republic of Korea

[6] KU Convergence Science and Technology Institute, Department of Stem Cell and Regenerative Biology, Konkuk University, Seoul 05029, Republic of Korea

[7] Department of Neurology, Samsung Medical Centre, School of Medicine, Sungkyunkwan University, Seoul 06351, Republic of Korea

* Corresponding authors

Address for Correspondence 1: Oh Young Bang, MD, PhD

Department of Neurology, Samsung Medical Centre, Sungkyunkwan University,

81 Irwon-ro, Gangnam-gu, Seoul 06351, South Korea

Tel.: +82-2-3410-3599, Fax: +82-2-3410-0052

E-mail: ohyoung.bang@samsung.com

Address for Correspondence 2: Jinseok Kim, PhD

Centre for Bionics, Biomedical Research Institute, Korea Institute of Science and Technology,

5 Hwarang-ro 14-gil, Seongbuk-gu, Seoul 02792, South Korea

Tel.: +82-2-958-6745, Fax: +82-2-958-6446

E-mail: jinseok@kist.re.kr

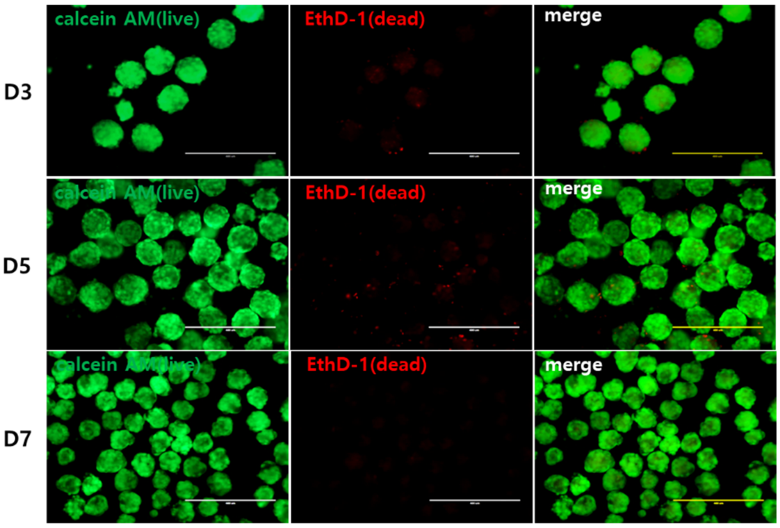

Supplementary Figure 1 A live (green) and dead (red) assay of cells in the 3D w/shaking group on D3, D5, and D7. Most cells in the hMSC-spheroids were highly viable. Initially, the hMSC-spheroids were homogeneously sized at approximately 150 μm in diameter, and as the culture continued, structural compaction of cellular aggregates progressed naturally. The size bars indicate 400 μm.

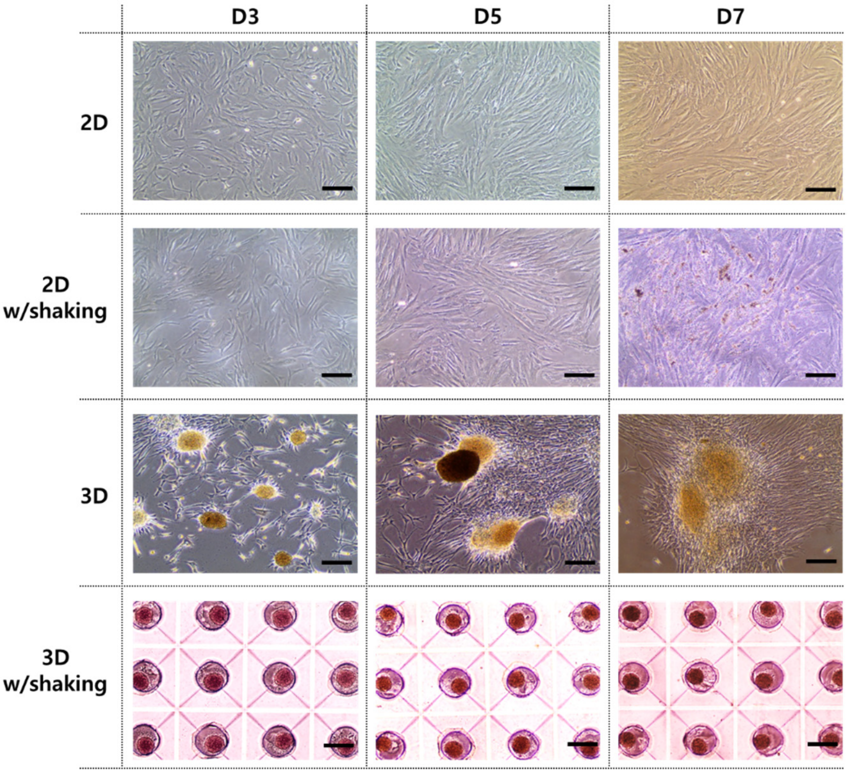

Supplementary Figure 2 Microscopic observation of cellular growth in the groups of 2D, 2D w/shaking, 3D, and 3D w/shaking over the culture period. The size bars indicate 200 μm.

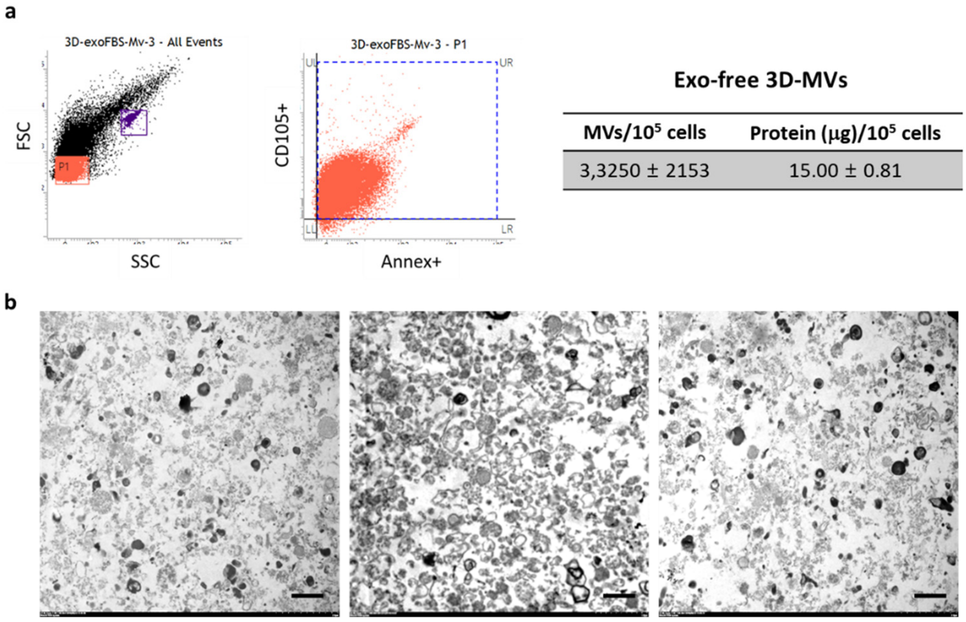

Exo-free 3D-MVs

MVs/10^5 cells	Protein (µg)/10^5 cells
3,3250 ± 2153	15.00 ± 0.81

Supplementary Figure 3 (a) A flow cytometric analysis for phenotyping and enumerating Exo-free 3D-MVs collected on D7. Particles sized below 1.0 µm (red solid squares), estimated using standard size beads, and those double-positive for anti-CD105 (hMSC surface marker) and anti-annexin V (lipid surface marker) were counted as hMSC-derived MVs (blue dotted squares). Counting beads (purple solid squares) were used to calculate the absolute counts of MVs. Quantitative comparison of counted MVs as normalized to the cell numbers on D7. A BCA protein quantification assay with Exo-free 3D-MVs collected on D7 which were normalized to the cell number. (b) TEM images of Exo-free 3D-MVs. Collected MVs revealed vesicular structures appearing rounded and bi-lipid layered, although differing in contrast and surface pattern. The size bars indicate 1,000 nm.

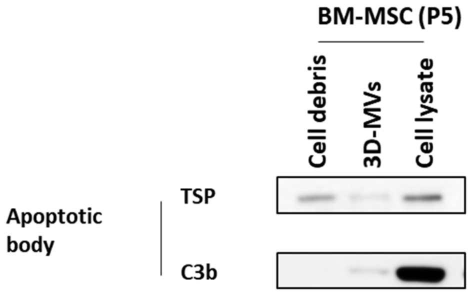

Supplementary Figure 4 Western blot analysis for markers of apoptotic bodies. Three samples were analysed: 1) our 3D-MVs, 2) cell debris collected during isolation of the 3D-MVs, and 3) cell lysate obtained from the common cell lysis procedure using hMSCs. The results showed that our 3D-MVs expressed minimal levels of thrombospondin (TSP) and C3b, while cell debris apparently expressed TSP and cell lysate expressed much higher levels of both markers.

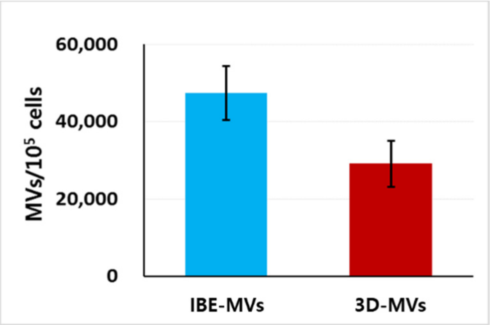

Supplementary Figure 5 Highly augmented production of hMSC-derived MVs with IBE treatment. Data are presented as the mean ± SEM.

a

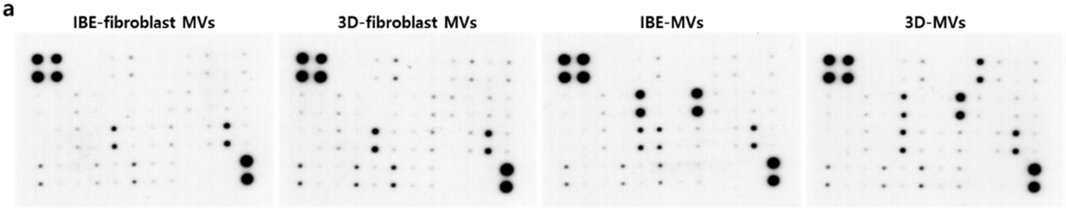

POSITIVE	POSITIVE	NEGATIVE	NEGATIVE	EOTAXIN	EOTAXIN-2	GCSF	GM-CSF	ICAM-1	IFN-γ	I-309	IL-1α
POSITIVE	POSITIVE	NEGATIVE	NEGATIVE	EOTAXIN	EOTAXIN-2	GCSF	GM-CSF	ICAM-1	IFN-γ	I-309	IL-1α
IL-1β	IL-2	IL-3	IL-4	IL-6	IL-6sR	IL-7	IL-8	IL-10	IL-11	IL-12p40	IL-12p70
IL-1β	IL-2	IL-3	IL-4	IL-6	IL-6sR	IL-7	IL-8	IL-10	IL-11	IL-12p40	IL-12p70
IL-13	IL-15	IL-16	IL-17	IP-10	MCP-1	MCP-2	M-CSF	MIG	MIP-1α	MIP-1β	MIP-1δ
IL-13	IL-15	IL-16	IL-17	IP-10	MCP-1	MCP-2	M-CSF	MIG	MIP-1α	MIP-1β	MIP-1δ
RANTES	TGF-β1	TNF-α	TNF-β	sTNF RI	sTNF RII	PDGF-BB	TIMP-2	BLANK	BLANK	NEGATIVE	POSITIVE
RANTES	TGF-β1	TNF-α	TNF-β	sTNF RI	sTNF RII	PDGF-BB	TIMP-2	BLANK	BLANK	NEGATIVE	POSITIVE

expressed by all

higher in IBE-MVs

higher in 3D-MVs

higher in both IBE- and 3D-MVs

b

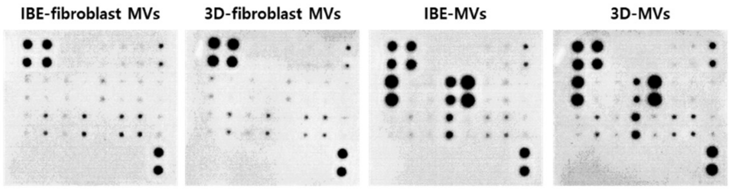

positive	positive	negative	negative	ANG	EGF	ENA-78	Bfgf
positive	positive	negative	negative	ANG	EGF	ENA-78	Bfgf
GRO	IFN gamma	IGF-1	IL-6	IL-8	Leptin	MCP-1	PDGF-BB
GRO	IFN gamma	IGF-1	IL-6	IL-8	Leptin	MCP-1	PDGF-BB
PLGF	RANTES	TGF beta1	TIMP-1	TIMP-2	THPO	VEGF	VEGF-D
PLGF	RANTES	TGF beta1	TIMP-1	TIMP-2	THPO	VEGF	VEGF-D
blank	blank	blank	blank	blank	blank	negative	positive
blank	blank	blank	blank	blank	blank	negative	positive

expressed by all

higher in IBE-MVs

higher in 3D-MVs

higher in both IBE- and 3D-MVs

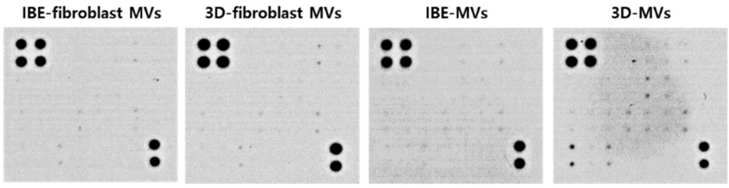

positive	positive	negative	negative	ANGPT-1	ANGPT-2	PLG	Endostatin
positive	positive	negative	negative	ANGPT-1	ANGPT-2	PLG	Endostatin
G-CSF	GM-CSF	I-309	IL-10	IL-1 alpha	IL-1 beta	IL-2	IL-4
G-CSF	GM-CSF	I-309	IL-10	IL-1 alpha	IL-1 beta	IL-2	IL-4
I-TAC	MCP-3	MCP-4	MMP-1	MMP-9	PECAM-1	TIE-2	TNF alpha
I-TAC	MCP-3	MCP-4	MMP-1	MMP-9	PECAM-1	TIE-2	TNF alpha
uPAR	VEGFR2	VEGFR3	blank	blank	blank	negative	positive
uPAR	VEGFR2	VEGFR3	blank	blank	blank	negative	positive

expressed by all

higher in MVs

higher in MVs

higher in both IBE- and 3D-MVs

c

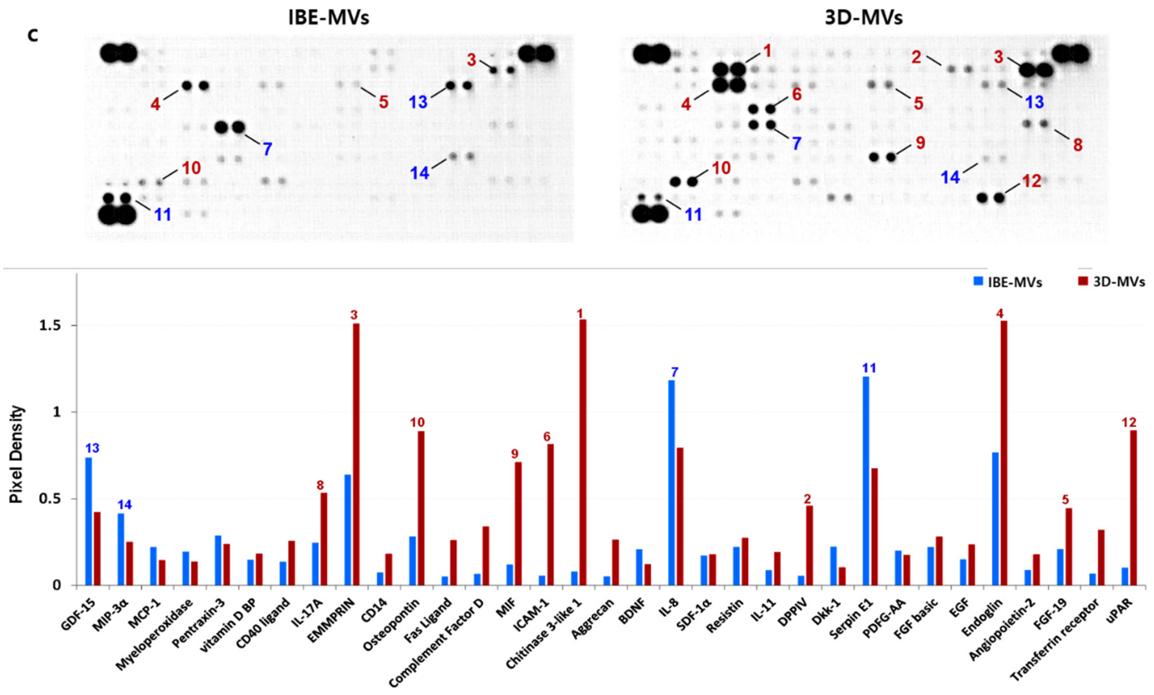

Supplementary Figure 6 High-throughput screening of various cytokines contained in MVs from IBE-treated fibroblasts (IBE-fibroblast MVs), dynamic 3D-cultured fibroblasts (3D-fibroblast MVs), IBE-treated hMSCs (IBE-MVs), and dynamic 3D-cultured hMSCs (3D-MVs). (a) Human Inflammation Antibody Array. (b) Human Angiogenesis Antibody Array. (c) Proteome Profiler™ Human XL Cytokine Array Kit. As compared to MVs generated from fibroblast cultures, those collected from hMSC cultures exclusively contained a variety of cytokines with therapeutic potential.

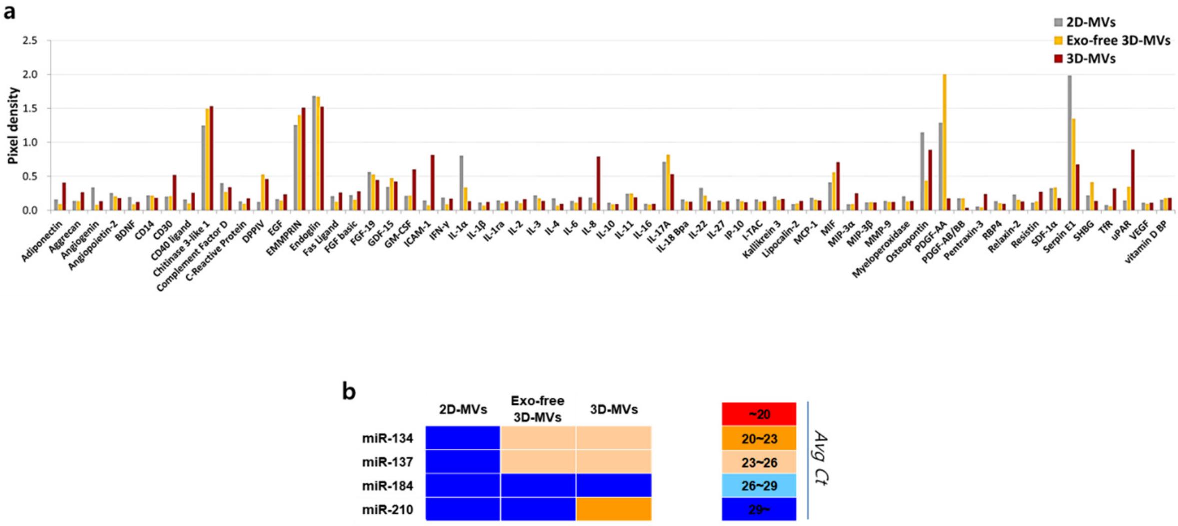

Supplementary Figure 7 (a) Representative cytokines contained in 2D-MVs, Exo-free 3D-MVs, and 3D-MVs were analysed using the Proteome Profiler^{TM} Human XL Cytokine Array Kit. Regardless of the variations in major compounds among groups, collected MVs abundantly contained various therapeutic cytokines. (b) micro-RNAs, such as miR-134, -137, -184, and -210, in 2D-MVs, Exo-free 3D-MVs, and 3D-MVs groups were analysed by the qPCR assay. miR-210 was minimally expressed in the Exo-free 3D-MVs group, unlike the 3D-MVs group. On the other hand, no expression of the selected micro-RNAs was detected in the 2D-MVs group.

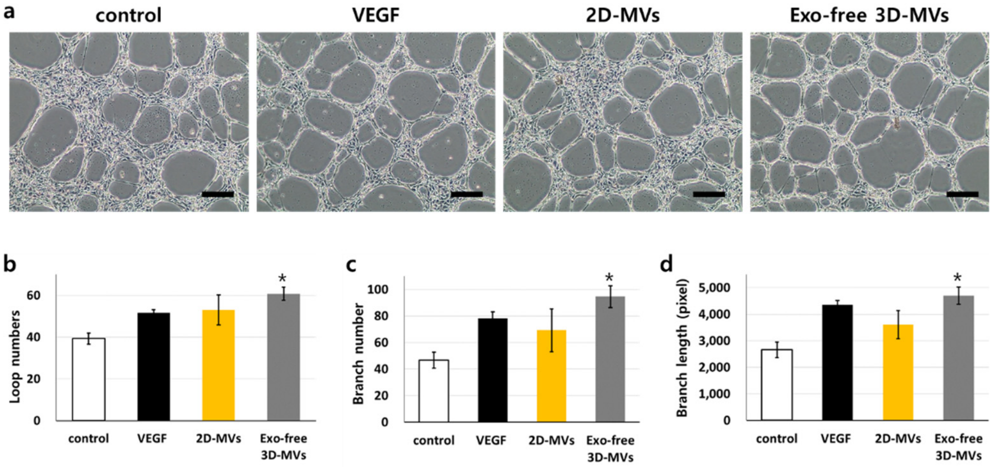

Supplementary Figure 8 Angiogenic stimulation via MV supplementation. (a) The inducible capacity of MVs for vascular tube formation. 3 μg/mL of 2D-MVs and Exo-free 3D-MVs were added to HUVECs plated on Matrigel, and resulting tube formation was observed along with a control (basal medium) and VEGF-treated groups by microscopy. The size bars indicate 200 μm. (b) Loop number (c) branch number and (d) branch length values of the resulting tube formation were quantitatively compared. Data are presented as the mean ± SEM. Differences among groups were evaluated by one-way ANOVA at a level of significance of $p < 0.05$ (*).

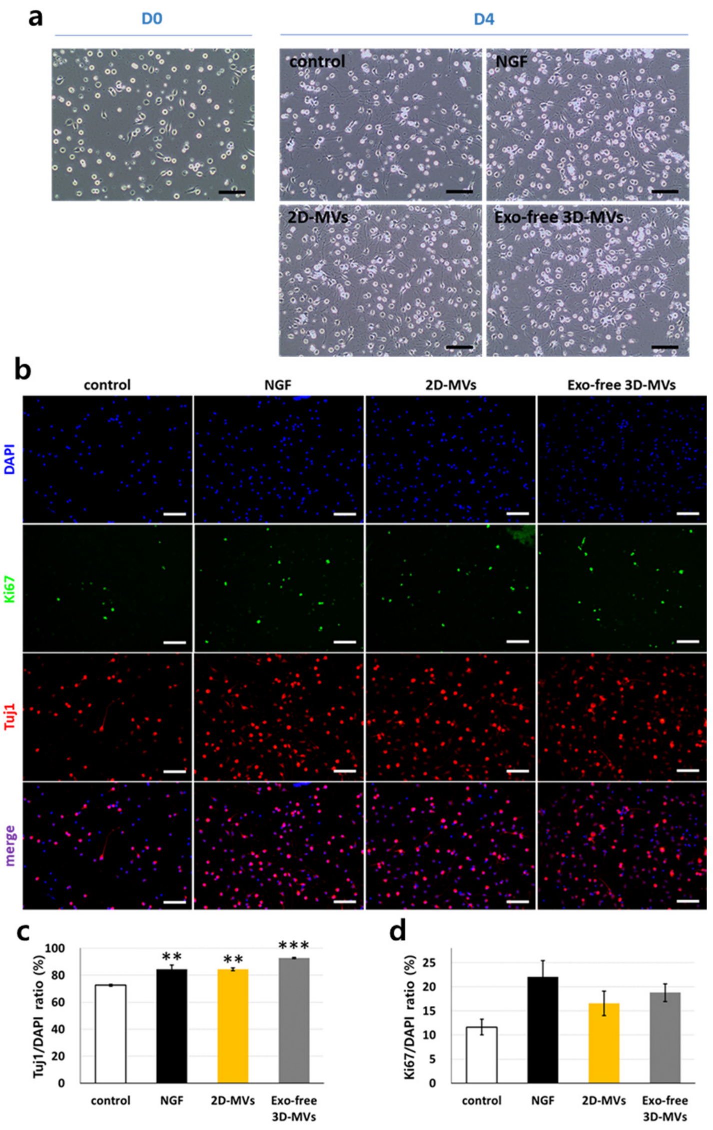

Supplementary Figure 9 Neurogenic stimulation via MV supplementation. (a) Phase contrast images of neurogenic stimulation of MVs. 3 μg/mL of 2D-MVs and Exo-free 3D-MVs were added to primarily cultured NSCs, and neural differentiation was observed on D4, compared with control (basal medium) and NGF-treated groups. The size bars indicate 100 μm. (b) Fluorescent image analyses of Tuj1 expression in NSCs along with Ki67 expression that denotes proliferating cells. The size bars indicate 100 μm. (c) Stimulated neurogenic differentiation of NSCs on D4 was quantified by counting cells positive for Tuj1 and normalized to DAPI-stained cells. (d) Proliferating NSCs on D4 were quantified by counting cells positive for Ki67 and normalized to DAPI-stained cells. Data are presented as the mean ± SEM. Differences among groups were evaluated by one-way ANOVA at a level of significance of $p < 0.001$ (***), $0.001 < p < 0.01$ (**), or $0.01 < p < 0.05$ (*).
